# Synthesis of 4,4′-(arylmethylene)bis(3-methyl-1-phenyl-1*H*-pyrazol-5-ols) and evaluation of their antioxidant and anticancer activities

**DOI:** 10.1186/s13065-021-00765-y

**Published:** 2021-06-03

**Authors:** José Eduardo Cadena-Cruz, Luis M. Guamán-Ortiz, Juan Carlos Romero-Benavides, Natalia Bailon-Moscoso, Kevin E. Murillo-Sotomayor, Nadia V. Ortiz-Guamán, Jorge Heredia-Moya

**Affiliations:** 1 Facultad de Ciencias Químicas, Universidad Central del Ecuador, Quito, Ecuador; 2 Departamento de Ciencias de La Salud, Universidad Técnica Particular de Loja, San Cayetano Alto s/n, C.P. 11 01 608, Loja, Ecuador; 3 Departamento de Química y Ciencias Exactas, Universidad Técnica Particular de Loja, San Cayetano Alto s/n, C.P. 11 01 608, Loja, Ecuador; 4 Centro de Investigación Biomédica (CENBIO), Facultad de Ciencias de la Salud Eugenio Espejo, Universidad UTE, 170527 Quito, Ecuador

**Keywords:** 4,4ʹ-(arylmethylene)bis(1*H﻿*-pyrazol-5-ols), Antioxidant, Apoptosis, Autophagy

## Abstract

**Background:**

Pyrazoles have attracted particular attention due to the diverse biological activities associated with this heterocyclic system, and some have been shown to be cytotoxic to several human cell lines. Several drugs currently on the market have this heterocycle as the key structural motif, and some have been approved for the treatment of different types of cancer.

**Results:**

4,4ʹ-(Arylmethylene)bis(1*H*-pyrazol-5-ols) derivatives **3a**–**q** were synthetized by a three components reaction of 3-methyl-1-phenyl-5-pyrazolone (**1**) with various benzaldehydes **2** catalyzed by sodium acetate at room temperature. The structures of all synthesized compounds were characterized by physicochemical properties and spectral means (IR and NMR) and were evaluated for their radical scavenging activity by DPPH assay and tested in vitro on colorectal RKO carcinoma cells in order to determine their cytotoxic properties. All 4,4ʹ-(arylmethylene)bis(1*H*-pyrazol-5-ols) derivatives **3a**–**q** were synthetized in high to excellent yield, and pure products were isolated by simple filtration. All compounds have good radical scavenging activity, and half of them are more active than ascorbic acid used as standard.

**Conclusion:**

Several derivatives proved to be cytotoxic in the RKO cell line. In particular, compound **3i** proved to be a very potent scavenger with an IC_50_ of 6.2 ± 0.6 µM and exhibited an IC_50_ of 9.9 ± 1.1 μM against RKO cell. Autophagy proteins were activated as a survival mechanism, whereas the predominant pathway of death was p53-mediated apoptosis.

**Supplementary Information:**

The online version contains supplementary material available at 10.1186/s13065-021-00765-y.

## Introduction

**Fig. 1:**
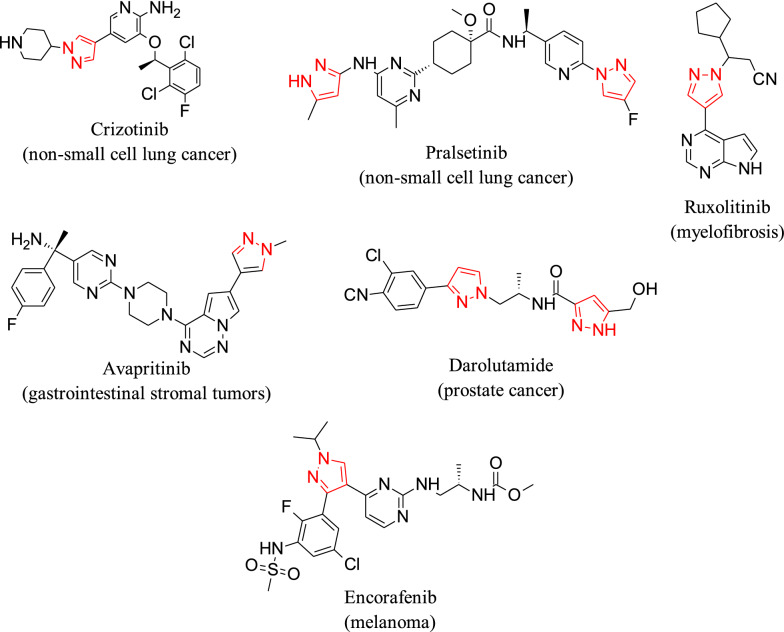
Approved anti-cancer pyrazole drugs

Heterocycles are common structural units in marketed drugs and in targets in the drug discovery process. Nitrogen-containing rings play an especially important role in drug development because of their wide variety of therapeutic and pharmacological properties [1]. Pyrazoles and their derivatives have attracted particular attention because they have a wide variety of biological activities [2, 3], and several drugs currently on the market, have the pyrazole ring as the key structural motif [4]. Some pyrazole derivatives have been demonstrated to be cytotoxic on several human cell lines [5–8], and, at this time, several drugs that have pyrazoles in their structure have been approved for the treatment of different types of cancer (see Fig. 1).

Edaravone, 3-methyl-1-phenyl-2-pyrazolin-5-one, (**1**) is a free radical scavenger approved for the treatment of amyotrophic lateral scleorosis (ALS) [9]. The compound is known to have preventive effects on myocardial injury following ischemia and reperfusion in patients with acute myocardial infarction and in brain edema after ischemia and reperfusion injury in animal models and in stroke patients [10]. There are several epidemiological studies related to the incidence of ALS and the development of cancer, that have reported the identification of a set of genes or signaling cascades involved in both diseases [11]. Also, it is well-known that many natural and synthesized antioxidants possessing phenolic hydroxyl groups have improved antioxidant activities by virtue of their abilities to react with free radicals [12], and studies carried out on flavones have shown that there is a relationship between antioxidant and anticancer activity [13].

Michael addition of an aromatic aldehyde **2** with an arylpyrazolone, obtained by the Knoevenagel reaction, allows an easy synthesis of 4,4ʹ-(arylmethylene) bis (1*H*-pyrazol-5-ols) **3**. These reactions can be done separately [14, 15] or in one step, either by a reaction of pseudo-five [16–18] or pseudo-three-components [19]. In practice, most of the reported synthetic routes consist of a one-step condensation of 3-methyl-1-phenyl-2-pyrazolin-5-one (**1**) with different aromatic aldehydes **2**, and most of the reactions used different types of catalyst. These edaravone derivatives incorporating hydroxyl groups in their structures, represent attractive targets for further study, however, only a few reports of biological activity were found in the literature, and the evaluation of compounds with hydroxyl groups is limited to only a few examples [20–23], and there is no report of cytotoxicity studies against cancer cells.

## Results and discussion

### Chemistry

**Scheme 1:**
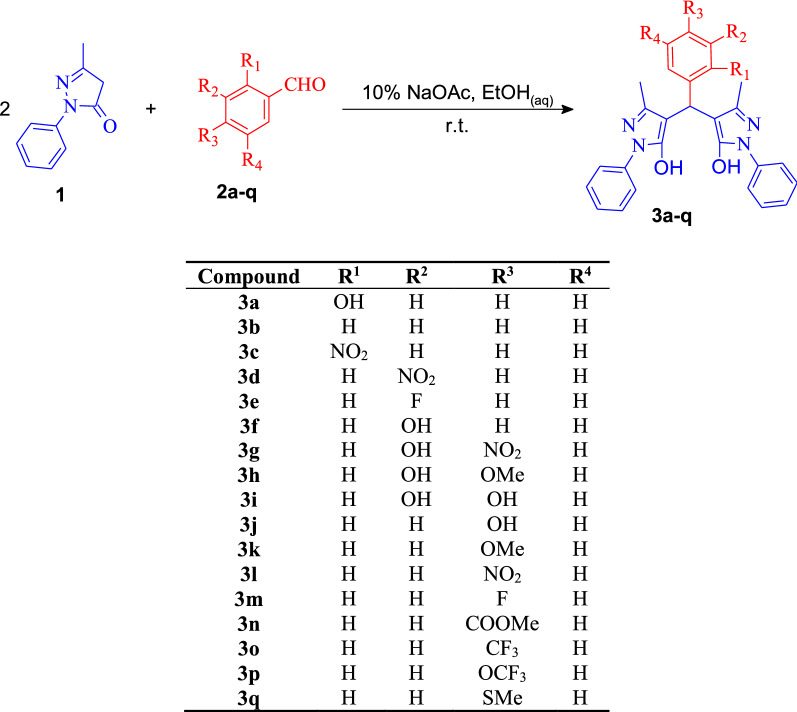
Synthesis of 4,4ʹ-(arylmethylene)bis(1*H*-pyrazol-5-ols) **3a**–**q**

The 4,4'-(arylmethylene)bis(1*H*-pyrazol-5-ols) **3** were synthesized using NaOAc as catalyst following the scheme depicted in Scheme 1, using 70% EtOH as solvent at room temperature. To find the optimal conditions, the reaction between **2b** and 2 equivalents of **1** was chosen as a model, showing 10% acetate gave the best catalytic effect.

With the optimized conditions, the scope of the reaction was studied using various substituted benzaldehydes **2a**–**q** bearing either electron-withdrawing or electron-donating groups to give the corresponding 4,4ʹ-bis-(arilmetilen)bis(1-fenil-3-metil-1*H*-pirazol-5-ol) derivatives **3a**–**q** in good to excellent yield as pure products by simple filtration. The spectroscopic data and melting points of compounds previously reported were in agreement with literature values (see Table 1 and “Experimental” section).

**Table 1:** Preparation of 4,4ʹ-(arylmethylene)bis(3-methyl-1-phenyl-1*H*-pyrazol-5-ols) derivatives **3a-q** catalyzed by 10% NaOAc at room temperature, DPPH scavenging activity and cytotoxic activity against RKO cell line

Compounds	Time (min)	Yield (%)	Mp (°C)	DPPH scavenging activity IC_50_ (µM)^a^	Cytotoxic activity IC_50_ (µM)
**3a**	10	98	219.5–220.6	17.1 ± 2.5	143.0 ± 4.9
**3b**	60	97	159.5–161.1	14.0 ± 2.3	105.9 ± 1.5
**3c**	10	95	210.0–211.0	20.9 ± 5.9	34.1 ± 4.4
**3d**	10	95^b^	150.7–152.0	16.6 ± 1.1	46.7 ± 3.3
**3e**	10	Quant	178.0–179.0	13.8 ± 0.7	36.3 ± 1.0
**3f**	480	98^c^	165.0–167.0	19.2 ± 1.5	84.3 ± 1.0
**3g**	40	99	202.0–204.0	18.7 ± 1.6	23.5 ± 5.9
**3h**	180	91^c^	200.0–202.0	13.7 ± 4.0	97.5 ± 3.0
**3i**	180	93^d^	182.7–184.0	6.2 ± 0.6^***^	9.9 ± 1.1
**3j**	180	97^c^	217.2–218.9	17.8 ± 3.7	77.8 ± 1.1
**3k**	120	92	176.0–177.0	19.7 ± 3.2	89.9 ± 3.0
**3l**	20	97	218.0–219.0	18.8 ± 3.8	27.3 ± 1.1
**3m**	60	87	183.8–185.8	10.2 ± 0.3	46.4 ± 1.0
**3n**	10	Quant	217.2–218.7	10.8 ± 0.3	36.0 ± 1.0
**3o**	15	96	203.0–205.0	9.8 ± 1.0^*^	23.6 ± 1.0
**3p**	60	Quant	174.5–176.0	12.3 ± 0.9	14.8 ± 1.0
**3q**	15	60	209.1–211.3	13.0 ± 1.0	40.9 ± 1.0
**Doxorubicin**	–	–	–	–	2.23 ± 0.02
**1**	–	–	–	18.1 ± 0.5	–
**Ascorbic acid**	–	–	–	14.0 ± 2.3	–

^a^The tests for significance were limited to ANOVA-Dunnett post-test, *p < 0.01, **p < 0.001, ***p < 0.0001 vs. **1**

^b^Using 100% EtOH

^c^Using 60% EtOH

^d^Using 50% EtOH

As expected, the time of the reaction with aldehydes bearing electron-withdrawing groups, independent of their location on the ring, were shorter than those with electron-donating groups, except with **2a**, which is six times shorter than benzaldehyde (entry 1). This could be due to the intramolecular hydrogen bonding in **2a** which enhances the reactivity of the aldehyde. When this hydrogen bond is lost, by protecting or changing the position of the hydroxyl group, the reaction time is higher. However, it is not clear the reason of the reduction of the activity observed in **3f**.

### Biological activity

All synthesized compounds were evaluated for antioxidant activity by the *N*,*N*-diphenyl-*N*ʹ-picrylhydrazyl (DPPH) assay as shown in Table 1. All compounds have good radical scavenging activity and half of them are more active than ascorbic acid used as standard. Compound **3c** has the lowest activity (20.9 µM) while compound **3i** proved to be a very potent scavenger with an IC_50_ of 6.2 µM. In fact, most bispyrazoles had better radical scavenging activity than **1**, and only **3c**, **3f**, **3k**, and **3l** are less potent scavengers.

Theoretical calculation of **1** shows that an H-atom abstraction rather than electron-transfer reaction is involved in the radical-scavenging process [24, 25]. Because in structure **3** the enol tautomer is more stable than the keto form, it would be expected that this hydrogen abstraction occurs on the hydroxyl group. Theoretical calculations of the enol tautomer of **1** show lower dissociation energy of this bond, so the abstraction of this hydrogen would be the most important for the scavenger properties of the edaravone [26].

The higher antioxidant activity of **3i** could suggest that the abstraction of the phenolic hydrogens instead of the enolic hydrogens would be more important for the stabilization of radicals, since the presence of the *ortho*-dihydroxy system is known to increase the stabilization of radicals [27]. This stabilization, provided by intramolecular hydrogen bonding in the radical formed has been confirmed by theoretical calculation in catechol derivatives [28]. Due to steric factors, the structure of **3** is not coplanar. Nonetheless, the results suggest that the conjugation of the radical is not restricted only to the pyrazol rings, since a contribution in the stabilization of the radical due to the aryl moiety is observed. Furthermore, both electron-donating and electron-withdrawing groups stabilize the radical, however, it is not clear how this stabilization occurs.

All derivatives were tested in vitro on colorectal RKO carcinoma cells in order to determine their cytotoxic properties (Table 1). Cells were exposed to each compound at five increasing concentrations for 48 h and their viability was monitored through MTS assay; as expected, dose-dependent effects were observed. For compounds **3a** and **3b** the observed IC_50_ was greater than 100 µM, however, the vast majority are below 50 µM, (**3c**, **3d**, **3e**, **3g**, **3i**, **3l**–**3q**), the compound with the highest cytotoxic activity was **3i** with an IC_50_ of 9.9 µM.

**Fig. 2:**
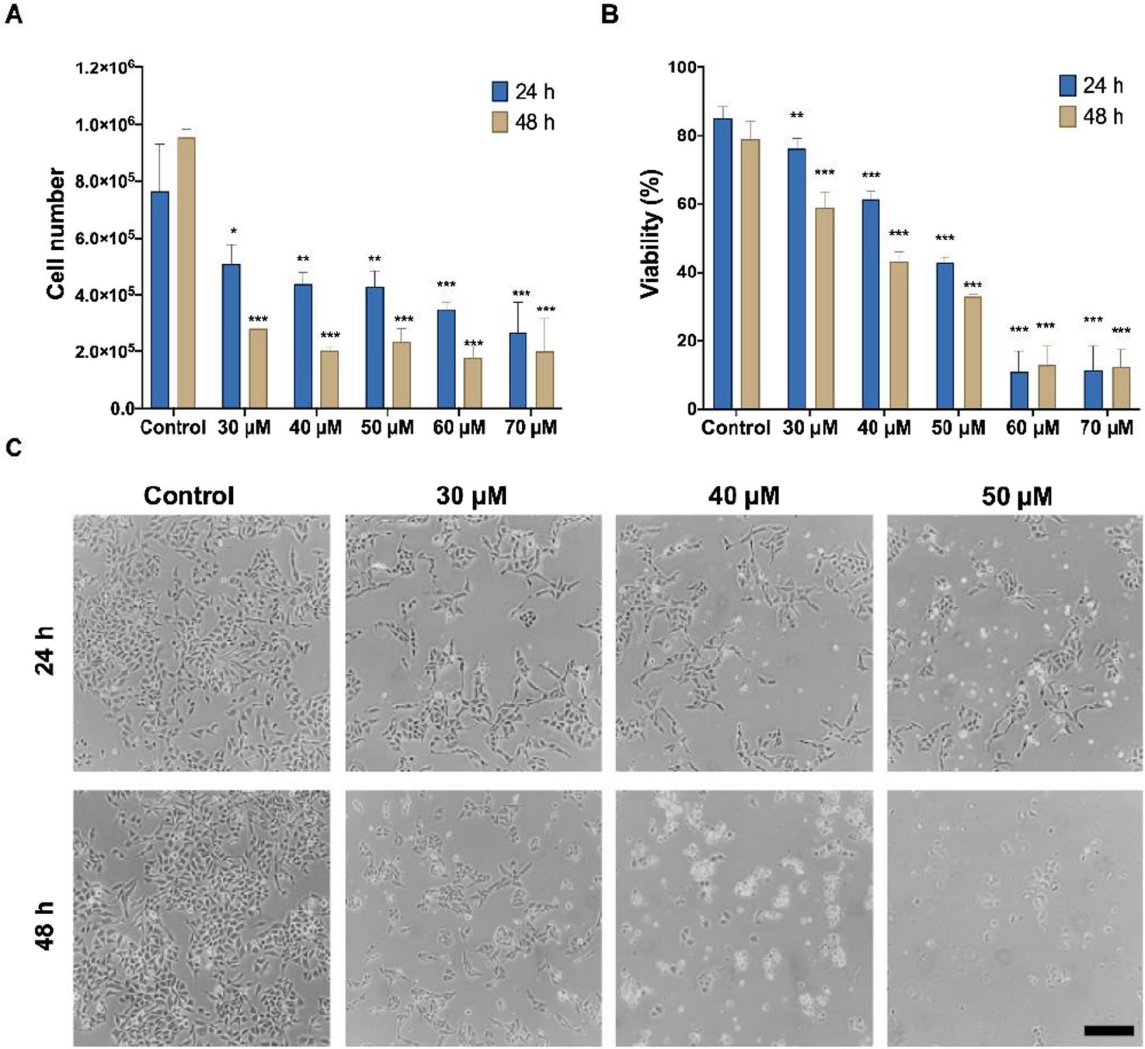
Cytotoxic effect of compound **3i**. RKO cell line were exposed to 10–70 µM of **3i** for 24 and 48 h and evaluated through Trypan Blue dye exclusion assay. **A** cell population, and **B** percentages of cell viability. The tests for significance were limited to ANOVA-Dunnett post-test, *p < 0.01, **p < 0.001, ***p < 0.0001 vs. control. **C** Cell morphology after exposure at 30, 40, and 50 µM; Horizontal bar in bottom figure = 50 µm

The MTS test is a proliferation test, but it does not distinguish between cytostatic and cytotoxic effect. To understand how the most powerful compound of the derivatives is acting, in this case **3i**, the trypan blue dye exclusion test was performed at 24 and 48 h. As indicated in Fig. 2, both cell numbers are seen to decrease in a dose-dependent manner (Fig. 2A); observing a decrease in the number of cells that could be related to a cytostatic effect. Thus, a decrease in cell viability related to the cytotoxic effect is also observed (Fig. 2B), the same as they agree with the morphological changes observed in Fig. 2C.

**Fig. 3:**
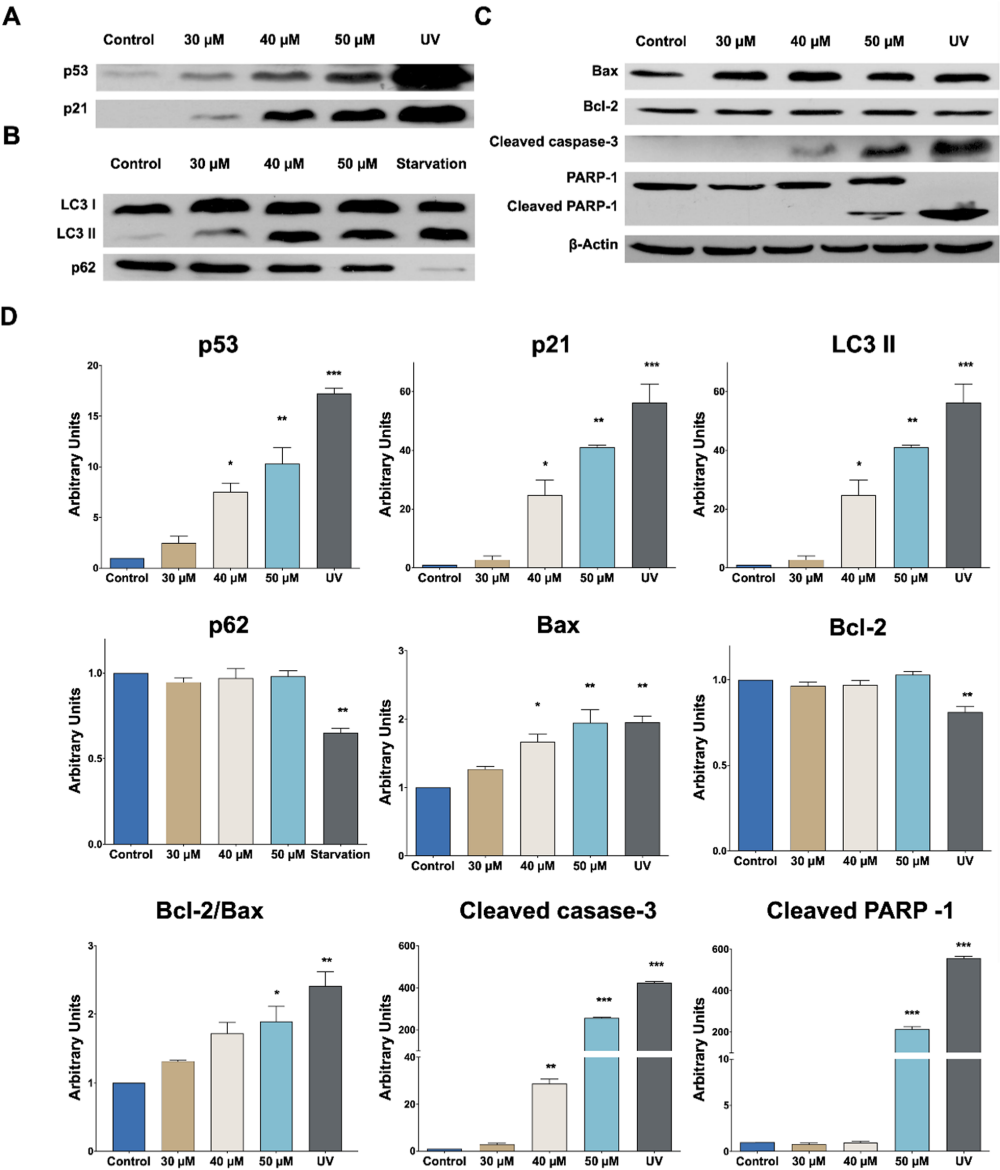
RKO cell line was exposed to different doses of compound **3i** for 24 h. Expression of protein: **A** p53 and p21 protein. **B** Pro-autophagic LC3 II and p62. **C** Pro-apoptotic proteins: Bax, Bcl-2, cleaved caspase-3, and PARP-1. Actin was used as control. **D** Quantification expression protein. The tests for significance were limited to ANOVA-Dunnet post-test: ∗ *p* < 0.01, ∗  ∗ *p* < 0.001, ∗  ∗  ∗ *p* < 0.0001 vs. control. Full-length gels are presented in Additional file 1: Fig. S1

Studied with the proteins involved in both apoptosis and autophagia, and p53 and p21 proteins controlling cell proliferation and death response were carried out [29]. Since cell death pathways must be examined prior to the morphological changes, a shorter time (24 h) and higher doses were chosen (30, 40 and 50 µM). Figure 3A shows that p53 increases significatively in a dose-dependent manner, indicating that this protein could be involved in the induced cell death process [30] by compound **3i**. A similar effect was observed in A2780 (ovarian adenocarcinoma), P388 (leukemia), and A549 (lung carcinoma) human cell lines [31, 32] after treatment with pyrazole derivatives. One of the major targets for p53 is p21, a protein related to cell cycle arrest; according to our results, p21 increases significantly in all doses tested (Fig. 3A, D); explaining therefore the inhibition of cell grow (cytostatic effect).

Autophagy pathway activation has been found to be induced by pyrazol derivatives in A549 lung cancer cell [33]. Therefore, pro-autophagic proteins were also studied (Fig. 3B, D). LC3 I is converted into its active form LC3 II at the beginning of the autophagy, while p62 is degraded in the last phase of this cell death pathway [34]. Our results showed a significant increase in LC3 II in the highest concentration, whereas no changes in p62 were detected. Accordingly, in our study, compound **3i** is able to induce autophagy as a protective mechanism [35]

It is well-known that p53 upregulates Bax to promote the intrinsic apoptosis pathway, which ends in the activation of caspase-3 [30]. Consequently, a significant increase in Bax upregulation was detected, together with the caspase-3 activation mostly in the highest concentration; whereas no changes were observed in the regulation of the anti-apoptotic protein Bcl-2 (Fig. 3C, D). This later protein has been found to interact with Beclin-1 to form the Bcl-2-Beclin-1 complex, which inhibits autophagy and allows the activation of apoptosis [36], thus explaining the observation that there is no variation in Bcl-2 expression and the lack of activation of autophagy. Moreover, a significant increase in Bax/Bcl-2 ratio was observed; indicating therefore that the RKO cell line is susceptible to apoptosis after treatment [37]. One of the multiple substrates of caspase-3 is PARP-1, a well-known apoptotic biomarker, which was also found significantly cleaved in the highest concentration (Fig. 3C, D). All these findings therefore show that compound **3i** is able to induce apoptosis on RKO cell line, mediated by p53.

Finally, the analysis of the substituents shows that compounds bearing electron-withdrawing groups, independent of their location on the ring, are more active than compounds with electron-donating substituents, except for **3i** (IC_50_ = 9.9 ± 1.1 μM) which is the most active compound despite having two hydroxyl groups. The *ortho* position of these hydroxyls would be responsible for the high activity observed since it has been reported that catechol compounds with hydroxyl residues *ortho* to each other are susceptible to oxidation leading to cell apoptosis, mainly due to generation of quinone through autoxidation and subsequent induction of p53 and caspase-3 activation [38].

## Conclusion

In this work, 4,4ʹ-(arylmethylene)bis(3-methyl-1-phenyl-1*H*-pyrazol-5-ols) **3a**–**q** were synthesized at room temperature using NaOAc as a catalyst in high to excellent yields and pure products were isolated by simple filtration. Most compounds show better DPPH radical scavenging activities than edaravone **1**, and some compounds show moderate cytotoxicity against RKO cell. In both activities the most potent compound was **3i**, with cytotoxic activity to the RKO cell line present in a dose and time-depended manner. Indeed, this compound was able to induce the apoptotic cell death pathway.

## Experimental

### Chemistry

All solvents and reagents were from Sigma Aldrich and used without further purification. All melting points are uncorrected and were determined on a Büchi Melting Point M-560 apparatus. FTIR spectra were recorded by a Perkin Elmer FTIR Spectrum One by using ATR system (4000–650 cm^−1^). The ^1^H and ^13^C NMR spectra were recorded at 298 K on a JEOL ECA 400 MHz or Bruker Advance 500 MHz spectrometer equipped with a z-gradient, triple-resonance (^1^H, ^13^C, ^15^N) cryoprobe, using DMSO-*d6* as solvent. The ^19^F-NMR spectra were acquired on an Oxford Instruments Pulsar benchtop NMR 60 MHz Spectrometer. Chemical shifts are expressed in ppm with tetramethylsilane (TMS, δ = 0 ppm) as an internal reference for protons and trifluoroacetic acid (TFA, δ = − 75.39 ppm) for fluorine. Accurate mass data were obtained using a Waters (Waltham, MA) model LCT Premiere time-of-flight (TOF) mass spectrometer. Reactions were monitored by TLC on silica gel using ethyl acetate/hexane mixtures as a solvent and compounds visualized by UV lamp. The reported yields are for the purified material and are not optimized.

### General procedure for the synthesis of 4,4′-(arylmethylene)bis(3-methyl-1-phenyl-1*H*-pyrazol-5-ols) 3a–q

To a solution of 0.4 mmol aldehyde **2a**–**q** and 0.8 mmol pyrazole **1** in 4 mL of 70% EtOH at room temperature, 40.2 μL of 1 M NaOAc were added and the mixture was stirred until the reaction was complete (see Table 1). Water was added to obtain 50% EtOH and the mixture was filtered, washed with 50% EtOH and dried to obtain pure product.

#### 4,4ʹ-[(2-Hydroxyphenyl)methylene]bis(3-methyl-1-phenyl-1H-pyrazol-5-ol) 3a

Yield 98% as a white solid; mp 219.5–220.6 °C [Lit. 218–220 [39]]; ^1^H-NMR (400 MHz, *DMSO-d*_*6*_) δ: 14.30 (br. s., 1 H, OH), 12.38 (br. s., 1 H, OH), 9.51 (br. s., 1 H, OH), 7.70 (d, *J* = 8.0 Hz, 4 H, Ar–H), 7.56 (d, *J* = 7.6 Hz, 1 H, Ar–H), 7.43 (t, *J* = 7.6 Hz, 4 H, Ar–H), 7.24 (t, *J* = 6.7 Hz, 2 H, Ar–H), 6.97 (t, *J* = 7.6 Hz, 1 H, Ar–H), 6.75 (d, *J* = 7.9 Hz, 1 H, Ar–H), 6.71 (t, *J* = 7.6 Hz, 1 H, Ar–H), 5.18 (s, 1 H, CH), 2.29 (s, 6 H, CH_3_).

#### 4,4ʹ-(Phenylmethylene)bis(3-methyl-1-phenyl-1H-pyrazol-5-ol) 3b

Yield 97% as a white solid; mp 159.5–161.1 °C [Lit. 161–163 [40]]; ^1^H-NMR (400 MHz, *DMSO-d*_*6*_) δ: 13.94 (br. s., 1 H, OH), 12.44 (br. s., 1 H, OH), 7.71 (d, *J* = 7.9 Hz, 4 H, Ar–H), 7.44 (t, *J* = 7.9 Hz, 4 H, Ar–H), 7.31–7.21 (m, 6 H, Ar–H), 7.20–7.14 (m, 1 H, Ar–H), 4.96 (s, 1 H, CH), 2.32 (br. s., 6 H, CH_3_).

#### 4,4'-[(2-Nitrophenyl)methylene]bis(3-methyl-1-phenyl-1H-pyrazol-5-ol) 3c

Yield 95% as a yellow solid; mp 210.0–211.0 °C [Lit. 218–220 [41]]; ^1^H-NMR (400 MHz, *DMSO-d*_*6*_) δ: 13.35 (br. s., 1 H, OH), 12.60 (br. s., 1 H, OH), 7.72 (d, *J* = 7.9 Hz, 1 H, Ar–H), 7.66 (d, *J* = 7.9 Hz, 4 H, Ar–H), 7.62 (m, 2 H, Ar–H), 7.48 (m, 1 H, Ar–H), 7.43 (t, *J* = 7.9 Hz, 4 H, Ar–H), 7.25 (t, *J* = 7.3 Hz, 2 H, Ar–H), 5.43 (s, 1 H, CH), 2.24 (br. s., 6 H, CH_3_).

#### 4,4'-[(3-Nitrophenyl)methylene]bis(3-methyl-1-phenyl-1H-pyrazol-5-ol) 3d

Yield 95% as a white solid; mp 150.7–152.0 °C [Lit. 150–152 [42]]; ^1^H-NMR (400 MHz, *DMSO-d*_*6*_) δ: 13.87 (br. s., 1 H, OH), 12.64 (br. s., 1 H, OH), 8.09 (s, 1 H, Ar–H), 8.08 (d, *J* = 8.6 Hz, 1 H, Ar–H), 7.74 (d, *J* = 7.9 Hz, 1 H, Ar–H), 7.70 (d, *J* = 8.5 Hz, 4 H, Ar–H), 7.61 (t, *J* = 8.6, 7.9 Hz, 1 H, Ar–H), 7.45 (t, *J* = 7.9 Hz, 4 H, Ar–H), 7.26 (d, *J* = 7.4 Hz,, 2 H, Ar–H), 5.15 (s, 1 H, CH), 2.35 (br. s., 6 H, CH_3_).

#### 4,4'-[(3-Fluorophenyl)methylene]bis(3-methyl-1-phenyl-1H-pyrazol-5-ol) 3e

Yield quantitative as a white solid; mp 178.0–179.0 °C [Lit. 183–184 [40]]; ^1^H-NMR (500 MHz, *DMSO-d*_*6*_) δ: 7.70 (d, *J* = 8.0 Hz, 4H, Ar–H), 7.44 (t, *J* = 7.8 Hz, 4H, Ar–H), 7.29–7.36 (m, 1H, Ar–H), 7.21—7.28 (m, 2H, Ar–H), 7.10 (d, *J* = 8.2 Hz, 1H, Ar–H), 6.98–7.04 (m, 2H, Ar–H), 4.97 (s, 1H, CH), 2.31 (br. s., 6H, CH_3_).

#### 4,4'-[(3-Hydroxyphenyl)methylene]bis(3-methyl-1-phenyl-1H-pyrazol-5-ol) 3f

Yield 98% as a white solid; mp 165.0–167.0 °C [Lit. 164–166 [43]]; ^1^H-NMR (400 MHz, *DMSO-d*_*6*_) δ: 13.95 (br. s., 1H, OH), 9.21 (s, 1H, Ar–H), 7.71 (d, *J* = 7.9 Hz, 4H, Ar–H), 7.44 (t, *J* = 7.8 Hz, 4H, Ar–H), 7.24 (t, *J* = 7.3 Hz, 2H, Ar–H), 7.04 (t, *J* = 7.8 Hz, 1H, Ar–H), 6.68 (br. s, 1H, OH), 6.65 (d, *J* = 7.7 Hz, 1H, Ar–H), 6.55 (dd, *J* = 7.9, 1.5 Hz, 1H, Ar–H), 4.86 (s, 1H), 2.30 (br. s., 6H).

#### 4,4'-[(3-Hydroxy-4-nitrophenyl)methylene]bis(3-methyl-1-phenyl-1H-pyrazol-5-ol) 3g

Yield 99% as a yellow solid; mp 202.0–204.0 °C (d); FTIR (cm^−1^): 3616 (OH), 1604 (C=C), 1600 (C=N), 1577 (NO_2_), 1499 (C=C), 1353 (OH), 1331 (NO_2_), 1233 (OH), 1120 (C–OH); ^1^H-NMR (500 MHz, *DMSO-d*_*6*_) δ: 13.84 (br. s., 1 H, OH), 10.84 (s, 1 H, OH), 7.83 (d, *J* = 8.2 Hz, 1 H, Ar–H), 7.71 (d, *J* = 8.2 Hz, 4 H, Ar–H), 7.45 (t, *J* = 8.0 Hz, 4 H, Ar–H), 7.26 (t, *J* = 7.4 Hz, 2 H, Ar–H), 7.04 (s, 1 H, Ar–H), 6.81 (dd, *J* = 1.9, 8.5 Hz, 1 H, Ar–H), 5.00 (s, 1 H, CH), 2.33 (br. s., 6 H, CH_3_); ^13^C-NMR (126 MHz, *DMSO-d*_*6*_) δ: 152.1, 150.5, 146.3, 134.7, 129.0, 125.7, 125.2, 120.6, 118.7, 118.4, 117.5, 33.0; ESI–MS m/z 497.8 [M]^+^.

#### 4,4'-[(3-Hydroxy-4-methoxyphenyl)methylene]bis(3-methyl-1-phenyl-1H-pyrazol-5-ol) 3h

Yield 91% as a white solid; mp 200.0–202.0 °C [Lit. 201–203 [44]]; ^1^H-NMR (500 MHz, *DMSO-d*_*6*_) δ: 13.90 (br. s., 1 H, OH), 12.38 (br. s., 1 H, OH), 8.82 (br. s., 1 H, OH), 7.71 (d, *J* = 8.23 Hz, 4 H, Ar–H), 7.44 (t, *J* = 7.68 Hz, 4 H, Ar–H), 7.24 (t, *J* = 6.86 Hz, 2 H, Ar–H), 6.79 (d, *J* = 8.78 Hz, 1 H, Ar–H), 6.69 (d, *J* = 1.65 Hz, 1 H, Ar–H), 6.59 (dd, *J* = 8.23, 1.65 Hz, 1 H, Ar–H), 4.83 (s, 1 H, CH), 3.70 (s, 3 H, OCH_3_), 2.30 (br. s., 6 H, CH_3_).

#### 4,4'-[(3,4-Dihydroxyphenyl)methylene]bis(3-methyl-1-phenyl-1H-pyrazol-5-ol) 3i

Yield 93% as a cream solid; mp 182.7–184.0 °C; FTIR (cm^−1^): 3206 (OH), 1598(C=N), 1568 (C=C), 1501 (C=C), 1368 (OH), 1291 (OH), 1191 (C-O); ^1^H-NMR (500 MHz, *DMSO-d*_*6*_) δ: 13.90 (br. s., 1 H, OH), 12.34 (br. s., 1 H, OH), 8.74 (s, 1 H, OH), 8.60 (br. s., 1 H, OH), 7.72 (d, *J* = 7.7 Hz, 4 H, Ar–H), 7.44 (t, *J* = 7.7 Hz, 4 H, Ar–H), 7.24 (t, *J* = 7.1 Hz, 2 H, Ar–H), 6.66 (d, *J* = 1.6 Hz, 1 H, Ar–H), 6.61 (d, *J* = 8.2 Hz, 1 H, Ar–H), 6.47 (dd, *J* = 1.6, 8.2 Hz, 1 H, Ar–H), 4.80 (s, 1 H, CH), 2.29 (br. s., 6 H, CH_3_); ^13^C-NMR (126 MHz, *DMSO-d*_*6*_) δ: 146.2, 144.8, 143.4, 132.9, 128.9, 125.5, 120.5, 117.8, 115.2, 114.8, 32.4, 11.6; HRMS (TOF ES +) *m/z* calcd for C_27_H_25_N_4_O_4_ (M+H)^+^: 469.1870; found: 469.1876.

#### 4,4'-[(4-Hydroxyphenyl)methylene]bis(3-methyl-1-phenyl-1H-pyrazol-5-ol) 3j

Yield 97% as a white solid; mp 217.2–218.9 °C [Lit. 214–216 [45]]; ^1^H-NMR (400 MHz, *DMSO-d*_*6*_) δ: 13.93 (br. s., 1H, OH), 9.17 (s, 1H, OH), 7.70 (d, *J* = 7.7 Hz, 4H, Ar–H), 7.43 (t, *J* = 7.8 Hz, 4H, Ar–H), 7.24 (t, *J* = 7.3 Hz, 2H, Ar–H), 7.04 (d, *J* = 8.4 Hz, 2H, Ar–H), 6.65 (d, *J* = 8.6 Hz, 2H, Ar–H), 4.84 (s, 1H, CH), 2.29 (br. s., 6H, CH_3_).

#### 4,4'-[(4-Methoxyphenyl)methylene]bis(3-methyl-1-phenyl-1H-pyrazol-5-ol) 3k

Yield 92% as a white solid; mp 176.0–177.0 °C [Lit. 176–177 [46]]; ^1^H-NMR (400 MHz, *DMSO-d*_*6*_) δ: 13.92 (br. s., 1 H, OH), 12.39 (br. s., 1 H, OH), 7.70 (d, *J* = 7.9 Hz, 4 H, Ar–H), 7.44 (t, *J* = 7.3, 7.9 Hz, 4 H, Ar–H), 7.24 (d, *J* = 7.3 Hz, 2 H, Ar–H), 7.16 (d, *J* = 8.5 Hz, 2 H, Ar–H), 6.83 (d, *J* = 8.5 Hz, 2 H, Ar–H), 4.89 (s, 1 H, CH), 3.70 (s, 3 H, OCH_3_), 2.30 (br. s., 6 H, CH_3_).

#### 4,4'-[(4-Nitrophenyl)methylene]bis(3-methyl-1-phenyl-1H-pyrazol-5-ol) 3l

Yield 97% as a yellow solid; mp 218.0–219.0 °C [Lit. 219–220 [47]]; ^1^H-NMR (500 MHz, *DMSO-d*_*6*_) δ: 8.17 (d, *J* = 8.9 Hz, 2 H, Ar–H), 7.69 (dd, *J* = 1.0, 8.6 Hz, 4 H, Ar–H), 7.51 (d, *J* = 8.9 Hz, 2 H, Ar–H), 7.45 (t, *J* = 8.0 Hz, 4 H, Ar–H), 7.26 (t, *J* = 7.4 Hz, 2 H, Ar–H), 5.13 (s, 1 H, CH), 2.34 (s, 6 H, CH_3_).

#### 4,4'-[(4-Fluorophenyl)methylene]bis(3-methyl-1-phenyl-1H-pyrazol-5-ol) 3m

Yield 87% as a white solid; mp 183.8–185.8 °C [Lit. 181–183 [48]]; ^1^H-NMR (500 MHz, *DMSO-d*_*6*_) δ: 7.59—7.65 (m, 4H, Ar–H), 7.42 (dd, J = 8.3, 7.4 Hz, 4H, Ar–H), 7.24 (t, J = 7.2 Hz, 4H, Ar–H), 7.06 (t, J = 8.9 Hz, 2H, Ar–H), 4.91 (s, 1H, CH), 2.27 (s, 6H, CH_3_).

#### Methyl 4-[bis(5-hydroxy-3-methyl-1-phenyl-1H-pyrazol-4-yl)methyl]benzoate 3n

Yield quantitative as a white solid; mp 217.2–218.7 °C¸ FTIR (cm^−1^): 1720 (C=O), 1595 (C=N), 1569 (C=C), 1499 (C=C), 1286 (C-O), 1118 (C-O); ^1^H-NMR (400 MHz, CDCl_3_) δ: 7.89 (d, *J* = 8.2 Hz, 2H, Ar–H), 7.60 (d, *J* = 7.9 Hz, 4H, Ar–H), 7.29 (t, *J* = 7.7 Hz, 4H, Ar–H), 7.26 (d, *J* = 7.6 Hz, 2H, Ar–H), 7.12 (t, *J* = 7.4 Hz, 2H, Ar–H), 4.80 (s, 1H, CH), 3.86 (s, 3H, COOCH_3_), 2.12 (s, 6H, CH_3_); ^13^C-NMR (101 MHz, CDCl_3_) δ: 167.2, 146.8, 146.1, 129.9, 129.1, 128.5, 128.2, 128.1, 127.5, 126.4, 121.4, 52.2, 34.1, 12.0; ESI–MS m/z 494.8 [M]^+^.

#### 4,4'-[(4-Trifluoromethylphenyl)methylene]bis(3-methyl-1-phenyl-1H-pyrazol-5-ol) 3o

Yield 96% as a white solid; mp 203.0–205.0 °C; FTIR (cm^−1^): 1600 (C=N), 1583 (C=C), 1500 (C=C), 1320 (CF_3_), 1115 (CF_3_); ^1^H-NMR (400 MHz, *DMSO-d*_*6*_) δ: 13.88 (s, 1H, OH), 7.70 (d, *J* = 7.7 Hz, 4H, Ar–H), 7.65 (d, *J* = 8.3 Hz, 2H, Ar–H), 7.46 (d, *J* = 8.4 Hz, 2H, Ar–H), 7.44 (t, *J* = 8.1 Hz, 4H, Ar–H), 7.25 (t, *J* = 7.3 Hz, 2H, Ar–H), 5.06 (d, *J* = 8.4 Hz, 1H, CH), 2.33 (s, 6H, CH_3_); ^13^C-NMR (101 MHz, *DMSO-d*_*6*_) δ: 147.1, 146.3, 128.9, 128.1, 126.7 (q, *J* = 31.5 Hz), 125.7 (m), 125.1 (q, *J* = 4.0 Hz), 124.5 (q, *J* = 271.9 Hz), 120.6, 33.0, 11.6; ^19^F-NMR (56.17 MHz, *DMSO-d*_*6*_) δ: − 60.12 (s, CF_3_); ESI–MS m/z 504.8 [M]^+^.

#### 4,4'-[(4-Trifluoromethoxyphenyl)methylene]bis(3-methyl-1-phenyl-1H-pyrazol-5-ol) 3p

Yield quantitative as a white solid; mp 174.5–176.0 °C; FTIR (cm^−1^): 1596 (C=N), 1579 (C=C), 1501 (C=C), 1253 (C–OCF_3_), 1226 (CF_3_), 1167 (CF_3_); ^1^H-NMR (500 MHz, *DMSO-d*_*6*_) δ: 7.70 (d, *J* = 7.8 Hz, 4H, Ar–H), 7.44 (t, *J* = 7.8 Hz, 4H, Ar–H), 7.35 (d, *J* = 8.6 Hz, 2H, Ar–H), 7.27 (d, *J* = 8.5 Hz, 2H, Ar–H), 7.25 (t, *J* = 7.3 Hz, 2H, Ar–H), 5.00 (s, 1H, CH), 2.32 (br. s., 6H, CH_3_); ^13^C NMR (126 MHz, DMSO-*d*_6_) δ: 147.1, 146.8, 141.9, 137.4, 137.2, 129.5, 129.4, 126.4, 121.2, 33.0, 11.8; ^19^F-NMR (56.17 MHz, *DMSO-d*_*6*_) δ: − 56.02 (s, OCF_3_); ESI–MS m/z 520.8 [M]^+^.

#### 4,4'-[(4-Thiomethylphenyl)methylene]bis(3-methyl-1-phenyl-1H-pyrazol-5-ol) 3q

Yield 60% as a white solid; mp 209.1–211.3 °C [Lit. 205–207 [49]]; ^1^H-NMR (400 MHz, *DMSO-d*_*6*_) δ: 13.91 (br. s., 1H, OH), 7.70 (d, *J* = 7.9 Hz, 4H, Ar–H), 7.44 (t, *J* = 7.8 Hz, 4H, Ar–H), 7.24 (t, *J* = 7.3 Hz, 2H, Ar–H), 7.18 (s, 4H, Ar–H), 4.91 (s, 1H, CH), 2.42 (s, 3H, SCH_3_), 2.31 (br. s., 6H, CH_3_).

## Biological evaluation

### DPPH radical scavenging assay

The stock solutions of the compounds was prepared by dissolving **3a**–**q** in dimethylsulfoxide (DMSO) to a concentration of 4 mg/mL. The solution was, diluted with methanol until a concentration of 400 µg/mL was obtained and then used immediately.

The experimental procedure was adapted from the literature [50]. Briefly, 100 µL of a 0.2 mM methanol solution of DPPH (2, 2-diphenyl-1-picrylhydrazyl) radical were added to 100 µL of methanolic solutions of **3a**–**q** prepared as serial two-fold dilutions from the stock solution in 96-well microfilter plates. Standards and edaravone were also prepared in the same concentrations. The mixture was incubated in dark at room temperature for 30 min and the absorbance was read at 515 nm on a Cytattion 5 (BioTek) spectrophotometer.

The % DPPH scavenging activity was then calculated by using the following formula:

$$\% {\text{DPPH}}\;{\text{scavenging}} = 100*\left[ {\frac{{\left( {{\text{A}}_{{{\text{sample}} + {\text{DPPH}}}} - {\text{A}}_{{\text{sample blank}}} } \right)}}{{\left( {{\text{A}}_{{{\text{DPPH}}}} - {\text{A}}_{{{\text{solvent}}}} } \right)}}} \right]$$

The antioxidant activity of the compound was expressed as IC_50_, which is defined as the concentration that could scavenge 50% of the DPPH free radical. The IC_50_ values were calculated in GraphPad Prism 8.1.1 (GraphPad Software, Corp.) The results are given as a mean ± standard deviation (SD) of experiments done in triplicate.

### Cell culture

For biological studies, a human colorectal carcinoma RKO cell line (donated by Dra. Patricia Ostrosky, Instituto de Investigaciones Biomédicas, Departamento de Genética y Toxicología Ambiental, UNAM), with wild type p53 was used. Cells were cultured in RPMI-1640 medium supplemented with FBS 10% (Sigma Aldrich, USA), glutamine 2 mM (GIBCO-Thermo Fisher Scientific, USA), streptomycin 0.1 mg/mL, penicillin 100 U/mL, and amphotericin B 0.25 µg/mL; and maintained at 37 °C in a humidified atmosphere containing 5% CO_2_. Derivatives were dissolved in DMSO at a stock concentration of 20 mM. The final concentration of DMSO (< 1%, v/v) did not affect the cell growth in the different experiments performed.

### Cytotoxic assay

The effect of each compound on cell proliferation was evaluated by the MTS metabolic viability assay, measuring mitochondrial activity of live cells. For this purpose, 2 × 10^3^ cells in 100 µL per well were seeded in triplicate in 96-well plates. Twenty-four hours after seeding, cells were exposed to each one of the derivatives at increasing concentrations (5–250 µM) and incubated for 48 h. Later, 20 µL of Cell Titer 96 Aqueous One Solution cell proliferation reagent (Promega, USA) was added to each well containing the cells 4 h before finishing the treatment. Then, the absorbance was measured with a microplate spectrophotometer (Epoch 2—BioTek, USA) at 492 nm. Data obtained from untreated cells (control) were considered as 100% of the viability to normalize the absorbance of treated samples.

Trypan Blue dye exclusion assay was also performed to determine the cell number and viability after exposure to compound **3i**. Briefly: 3 × 10^4^ cells in 2 mL per well were seeded in a 6-well plate; after 24 h, cells were exposed to compound **3i** at 10–70 µM and incubated for additional 24 and 48 h. Supernatant from wells was recovered independently, cells were then trypsinized, collected, and mixed with the previously recovered medium. After centrifugation, pellets were re-suspended in 1 mL of fresh medium. Cell suspension was mixed with Trypan Blue 0.4% (GIBCO-Thermo Fisher Scientific, USA) in a 1:1 proportion and then counted applying a hemocytometer. Viable and non-viable (stained) cells were counted in a light microscopy (Nikon, USA).

For morphological analysis, 5 × 10^4^ cells/mL were seeded in 3.5-cm diameter Petri dishes. After 24 h of incubation, cells were exposed to compound **3i** at three representative doses: 30, 40 and 50 µM, for 24 and 48 h. Subsequently, cell morphology was observed using a light microscope (Axioskop 2 plus—Zeiss, Germany) equipped with a 40× objective. Images were then acquired with a SCA1300-32FM digital camera (Basler Inc., Germany).

### Western blot analysis

Western blot analyses were performed in order to determine the induced cell death pathway by derivatives. According to cytotoxic effect, three concentrations (30, 40 and 50 µM) were administrated to RKO cell line for 24 h. As positive controls, cells were simultaneously exposed for 10 min to UV irradiation (Osram, G30T8, 30 W Germicidal UV-C Lamp, 254 nm) for apoptosis induction or for 1 h to PBS for starvation-induced autophagy [51]. As follows, proteins were separated by SDS-PAGE (7–15%) and transferred to a PVDF membrane (IPVH00010, Immobilon-P, 0.45 µm, EMD/Millipore, Billerica, USA), and then incubated with primary antibodies: p53 (sc-81168), p21 (sc-817), SQSTM1/p62 (sc-48402), β-actin (sc-58673) (Santa Cruz Biotechnology, USA), PARP (#9542), caspase-3 (#6962), Bax (#2774), Bcl-2 (#15071) LC3A/B (#12741) (Cell Signaling Technology, USA), followed by secondary antibodies: anti-mouse IgG, HRP-linked (#7076) and anti-rabbit IgG, HRP-linked (#7074) (Cell Signaling Technology, USA). Immunoreactive bands were monitored using Immobilon Crescendo, or Forte, Western HRP Substrate (Millipore-Merck, KGaA, Germany).

### Statistical analysis

Each experiment was performed independently at least three times and data were reported as the means ± SEM, as evidenced in each figure. Significant data were obtained with one-way analysis of variance (ANOVA) followed by the Dunnett post-test. Samples exposed to derivatives, Doxorrubicin or UV or starvation condition were compared to the control considering a *p* < 0.05 to be statistically significant. Statistical analyses were realized in GraphPad Prism 8 (GraphPad Software, USA).

## Supplementary Information

**Additional file 1: Fig. S1**. Original western blots CL-X Posure™ films for Western blots. Each protein was detected in independent films at different times of exposure to the membrane. A) p53; B) p21; C) LC3-I and -II; D) p62; E) BAX; F) Bcl-2; G) Cleaved caspase-3; H) Active and cleaved PARP-1; I) Actin. Notice, for autophagy detection starvation control was first, in contrast to apoptosis control, which UV control was first. **Fig. S2.** ^1^H NMR spectrum of compound **3a**. **Fig. S3.** ^1^H NMR spectrum of compound **3b**. **Fig. S4.** ^1^H NMR spectrum of compound **3c**. **Fig. S5.** ^1^H NMR spectrum of compound **3d**. **Fig. S6.** ^1^H NMR spectrum of compound **3e**. **Fig. S7.** ^19^F NMR spectrum of compound **2e**. **Fig. S8.** ^1^H NMR spectrum of compound **3f**. **Fig. S9.** ^1^H NMR spectrum of compound **3 g**. **Fig. S10.** ^13^C NMR spectrum of compound **3 g**. **Fig. S11.** FTIR spectrum of compound **3 g**. **Fig. S12.** ESI–MS spectrum of compound **3 g**. **Fig. S13.** ^1^H NMR spectrum of compound **3 h**. **Fig. S14.** ^1^H NMR spectrum of compound **3i**. **Fig. S15.** ^13^C NMR spectrum of compound **3i**. **Fig. S16.** FTIR spectrum of compound **3i**. **Fig. S17.** HRMS spectrum of compound **3i**. **Fig. S18.** ^1^H NMR spectrum of compound **3j**. **Fig. S19.** ^1^H NMR spectrum of compound **3 k**. **Fig. S20.** ^1^H NMR spectrum of compound **3 l**. **Fig. S21.** ^1^H NMR spectrum of compound **3 m**. **Fig. S22.** ^19^F NMR spectrum of compound **3 m**. **Fig. S23.** ^1^H NMR spectrum of compound **3n**. **Fig. S24.** ^13^C NMR spectrum of compound **3n**. **Fig. S25.** FTIR spectrum of compound **3n**. **Fig. S26.** ESI–MS spectrum of compound **3n**. **Fig. S27.** ^1^H NMR spectrum of compound **3o**. **Fig. S28.** ^13^C NMR spectrum of compound **3o**. **Fig. S29.** ^19^F NMR spectrum of compound **3o**. **Fig. S30.** FTIR spectrum of compound **3o**. **Fig. S31.** ESI–MS spectrum of compound **3o**. **Fig. S32.** ^1^H NMR spectrum of compound **3p**. **Fig. S33.** ^13^C NMR spectrum of compound **3p**. **Fig. S34.** ^19^F NMR spectrum of compound **3p**. **Fig. S35.** FTIR spectrum of compound **3p**. **Fig. S36.** ESI–MS spectrum of compound **3p**. **Fig. S37.** ^1^H NMR spectrum of compound **3q**.

## Data Availability

All data generated or analysed during this study are included in this published article [and its additional information file].

## Abbreviations

FTIR: Fourier-transform infrared
NMR: Nuclear magnetic resonance
DPPH: 2,2-Diphenyl-1-picryl-hydrazyl-hydrate
RKO: Human colorectal carcinoma cell line
ALS: Amyotrophic lateral sclerosis
NaOAc: Sodium acetate
EtOH: Ethanol
IC_50_: Half maximal inhibitory concentration/median inhibitory concentration
µM: Micromolar
Mp: Melting point
min: Minutes
Quant: Quantitative
MTS: 3-(4,5-Dimethylthiazol-2-yl)-2,5-diphenyltetrazolium bromide
p53: Tumor protein p53
p21: Cyclin-dependent kinase inhibitor 1
A2780: Human ovarian adenocarcinoma cell line
P388: Human leukemia cell line
A549: Human lung carcinoma cell line
LC3 II: Microtubule-associated protein 1A/1B-light chain 3
LC3 I: Cytosolic form of LC3
p62: Ubiquitin-binding protein p62
Bax: Bcl-2-Like Protein 4
Bcl-2: B-cell lymphoma 2
PARP-1: Poly [ADP-ribose] polymerase 1
ATR: Attenuated total refection
DMSO: Dimethyl sulfoxide
TMS: Tetramethylsilane
TOF: Time of flight
TLC: Thin layer chromatography
UV: Ultraviolet
HRMS: High-resolution mass spectrometry
ES+: Positive electrospray ionization
RPMI: Cell culture media Roswell Park Memorial Institute
FBS: Fetal bovine serum
PBS: Phosphate-buffered saline
SDS-PAGE: Sodium dodecyl sulphate-polyacrylamide gel electrophoresis
PVDF: Polyvinylidene difluoride
SQSTM1/p62: Sequestosome-1
IgG: Immunoglobulin G
HRP: Horseradish peroxidase
SEM: Standard error of the mean
