# Supplementary material for: Synthesis of 4,4′-(arylmethylene)bis(3-methyl-1-phenyl-1H-pyrazol-5-ols) and evaluation of their antioxidant and anticancer activities
Source: BMC Chem. 2021 Jun 3;15(1):38. doi: 10.1186/s13065-021-00765-y (PMC8176600; doi:10.1186/s13065-021-00765-y)
Supplement: Supplementary file 1 — Additional file 1: Fig. S1. Original western blots CL-X Posure™ films for Western blots. Each protein was detected in independent films at different times of exposure to the membrane. A) p53; B) p21; C) LC3-I and -II; D) p62; E) BAX; F) Bcl-2; G) Cleaved caspase-3; H) Active and cleaved PARP-1; I) Actin. Notice, for autophagy detection starvation control was first, in contrast to apoptosis control, which UV control was first. Fig. S2. 1H NMR spectrum of compound 3a. Fig. S3. 1H NMR spectrum of compound 3b. Fig. S4. 1H NMR spectrum of compound 3c. Fig. S5. 1H NMR spectrum of compound 3d. Fig. S6. 1H NMR spectrum of compound 3e. Fig. S7. 19F NMR spectrum of compound 2e. Fig. S8. 1H NMR spectrum of compound 3f. Fig. S9. 1H NMR spectrum of compound 3 g. Fig. S10. 13C NMR spectrum of compound 3 g. Fig. S11. FTIR spectrum of compound 3 g. Fig. S12. ESI–MS spectrum of compound 3 g. Fig. S13. 1H NMR spectrum of compound 3 h. Fig. S14. 1H NMR spectrum of compound 3i. Fig. S15. 13C NMR spectrum of compound 3i. Fig. S16. FTIR spectrum of compound 3i. Fig. S17. HRMS spectrum of compound 3i. Fig. S18. 1H NMR spectrum of compound 3j. Fig. S19. 1H NMR spectrum of compound 3 k. Fig. S20. 1H NMR spectrum of compound 3 l. Fig. S21. 1H NMR spectrum of compound 3 m. Fig. S22. 19F NMR spectrum of compound 3 m. Fig. S23. 1H NMR spectrum of compound 3n. Fig. S24. 13C NMR spectrum of compound 3n. Fig. S25. FTIR spectrum of compound 3n. Fig. S26. ESI–MS spectrum of compound 3n. Fig. S27. 1H NMR spectrum of compound 3o. Fig. S28. 13C NMR spectrum of compound 3o. Fig. S29. 19F NMR spectrum of compound 3o. Fig. S30. FTIR spectrum of compound 3o. Fig. S31. ESI–MS spectrum of compound 3o. Fig. S32. 1H NMR spectrum of compound 3p. Fig. S33. 13C NMR spectrum of compound 3p. Fig. S34. 19F NMR spectrum of compound 3p. Fig. S35. FTIR spectrum of compound 3p. Fig. S36. ESI–MS spectrum of compound 3p. Fig. S37. 1H NMR spectrum of compound 3q. [file 13065_2021_765_MOESM1_ESM.pdf]

Supplementary Information

Synthesis of 4,4′-(arylmethylene)bis(3-methyl-1-phenyl-1*H*-pyrazol-5-ols) and evaluation of their antioxidant and anticancer activities

José Eduardo Cadena-Cruz [1], Luis M. Guamán-Ortiz [2], Juan Carlos Romero-Benavides[3], Natalia Bailon-Moscoso[2], Kevin E. Murillo-Sotomayor[2], Nadia V. Ortiz-Guamán[2], Jorge Heredia-Moya[4,]*

[1] *Facultad de Ciencias Químicas, Universidad Central del Ecuador, Quito, Ecuador*

[2] *Departamento de Ciencias de la Salud, Universidad Técnica Particular de Loja, San Cayetano Alto s/n, C.P. 11 01 608, Loja, Ecuador*

[3] *Departamento de Química y Ciencias Exactas, Universidad Técnica Particular de Loja, San Cayetano Alto s/n, C.P. 11 01 608, Loja, Ecuador*

[4] *Centro de Investigación Biomédica (CENBIO), Facultad de Ciencias de la Salud Eugenio Espejo,*

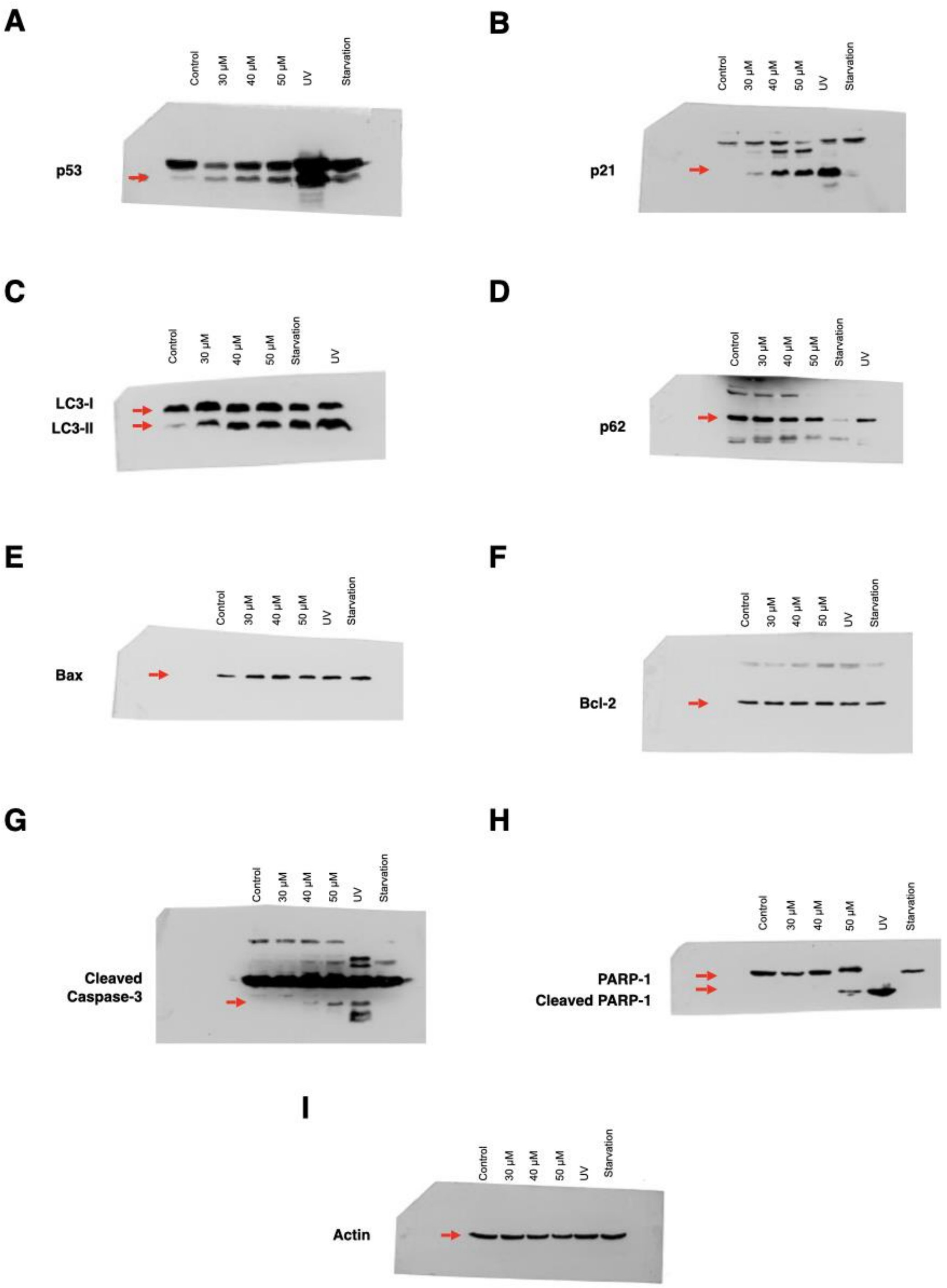

Fig. S1. Original western blots CL-X Posure™ films for Western blots. Each protein was detected in independent films at different times of exposure to the membrane. **A**) p53; **B**) p21; **C**) LC3-I and -II; **D**) p62; **E**) BAX; **F**) Bcl-2; **G**) Cleaved caspase-3; **H**) Active and cleaved PARP-1; **I**) Actin. Notice, for autophagy detection starvation control was first, in contrast to apoptosis control, which UV control was first.

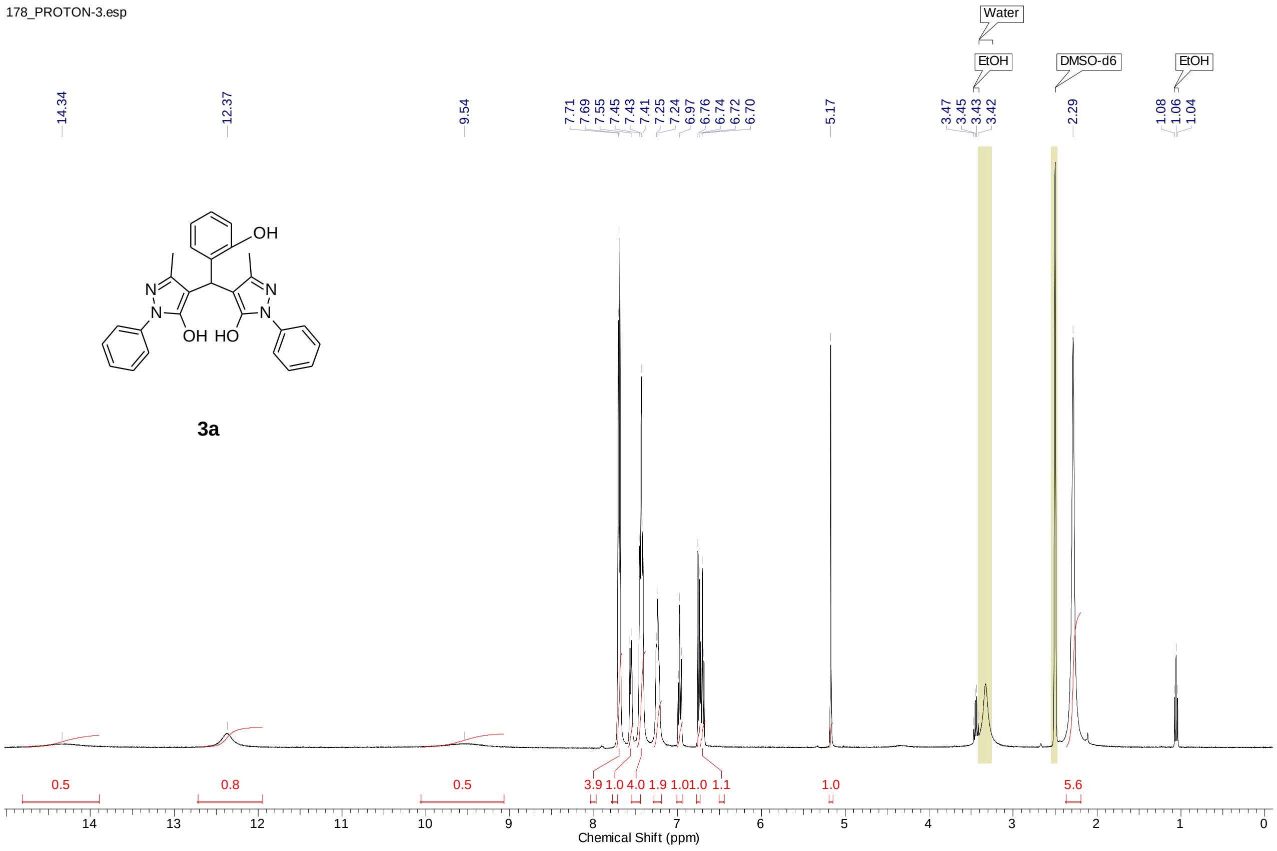

Fig. S2. ^{1}H NMR spectrum of compound **3a**.

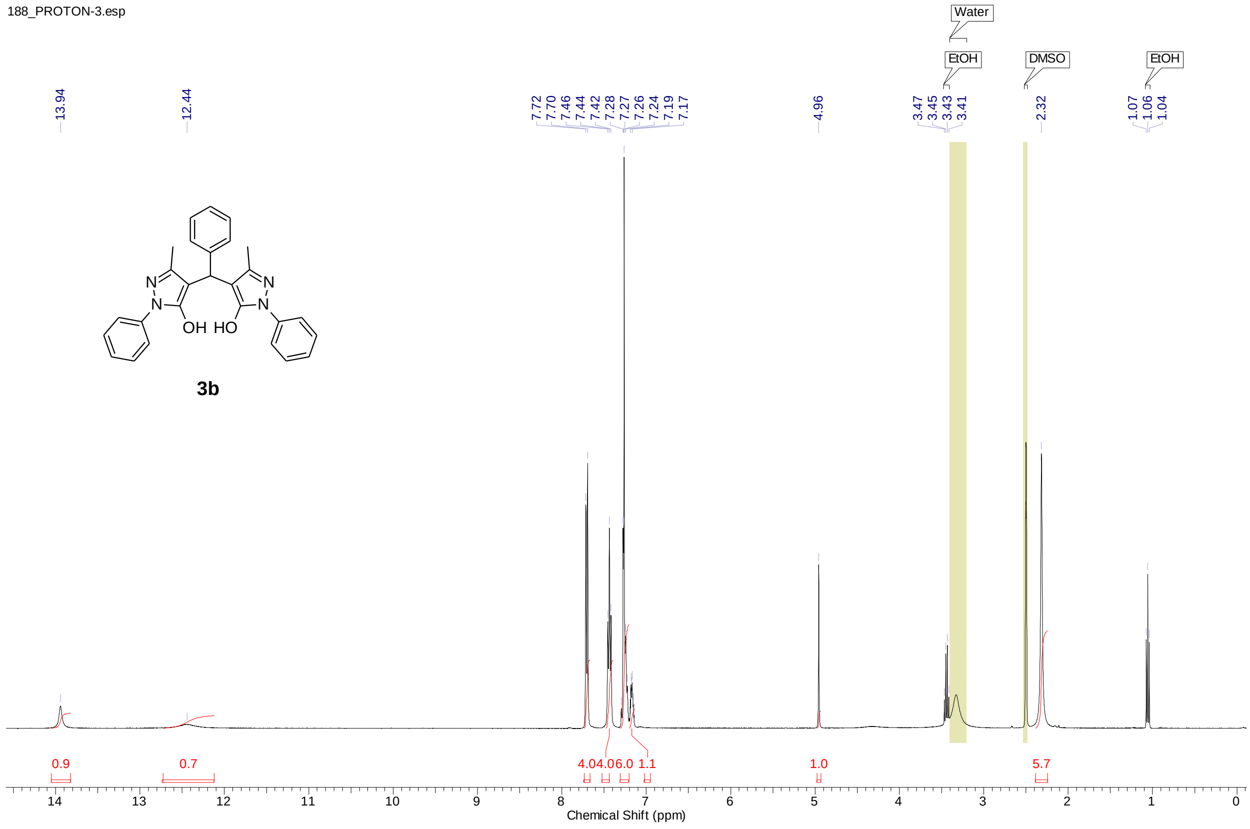

Fig. S3. ^{1}H NMR spectrum of compound **3b**.

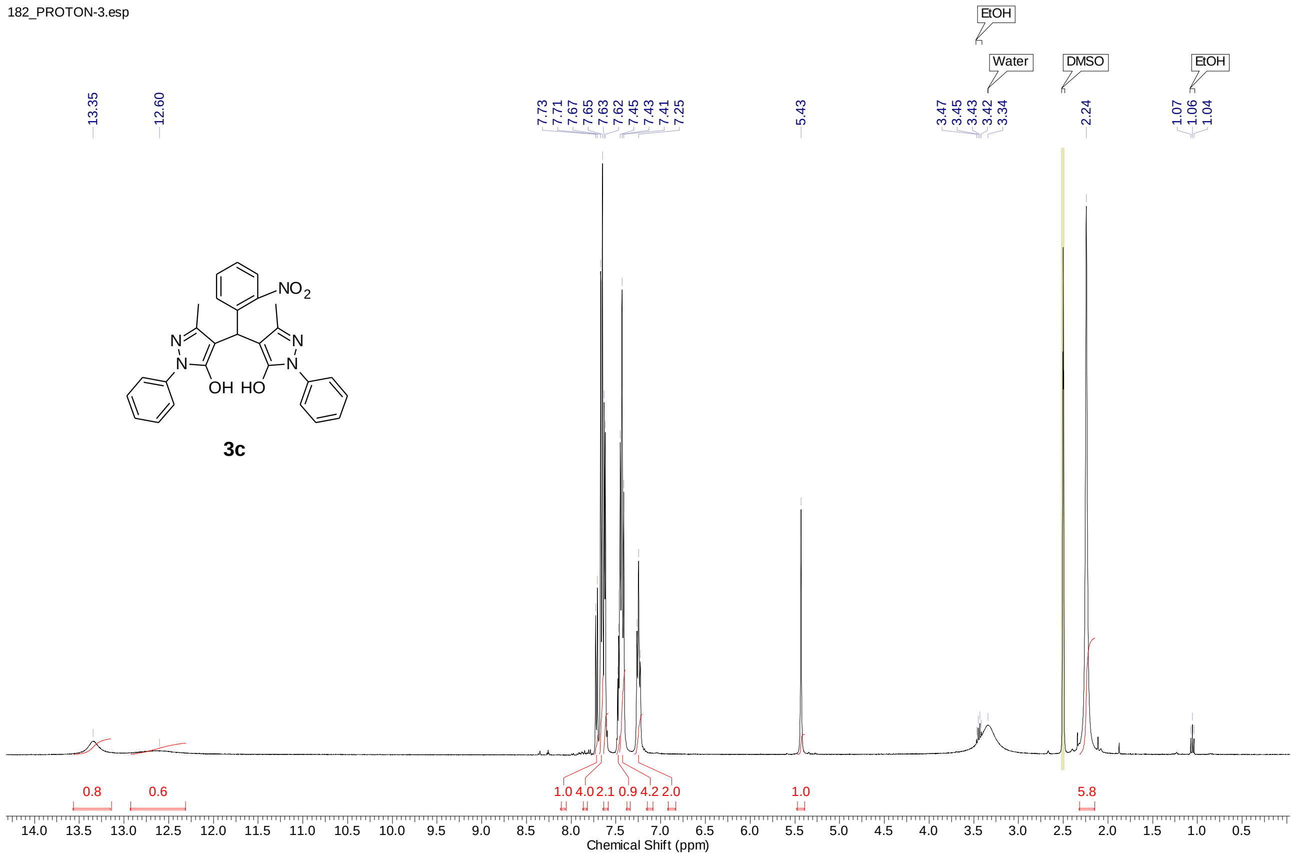

Fig. S4. ¹H NMR spectrum of compound **3c**.

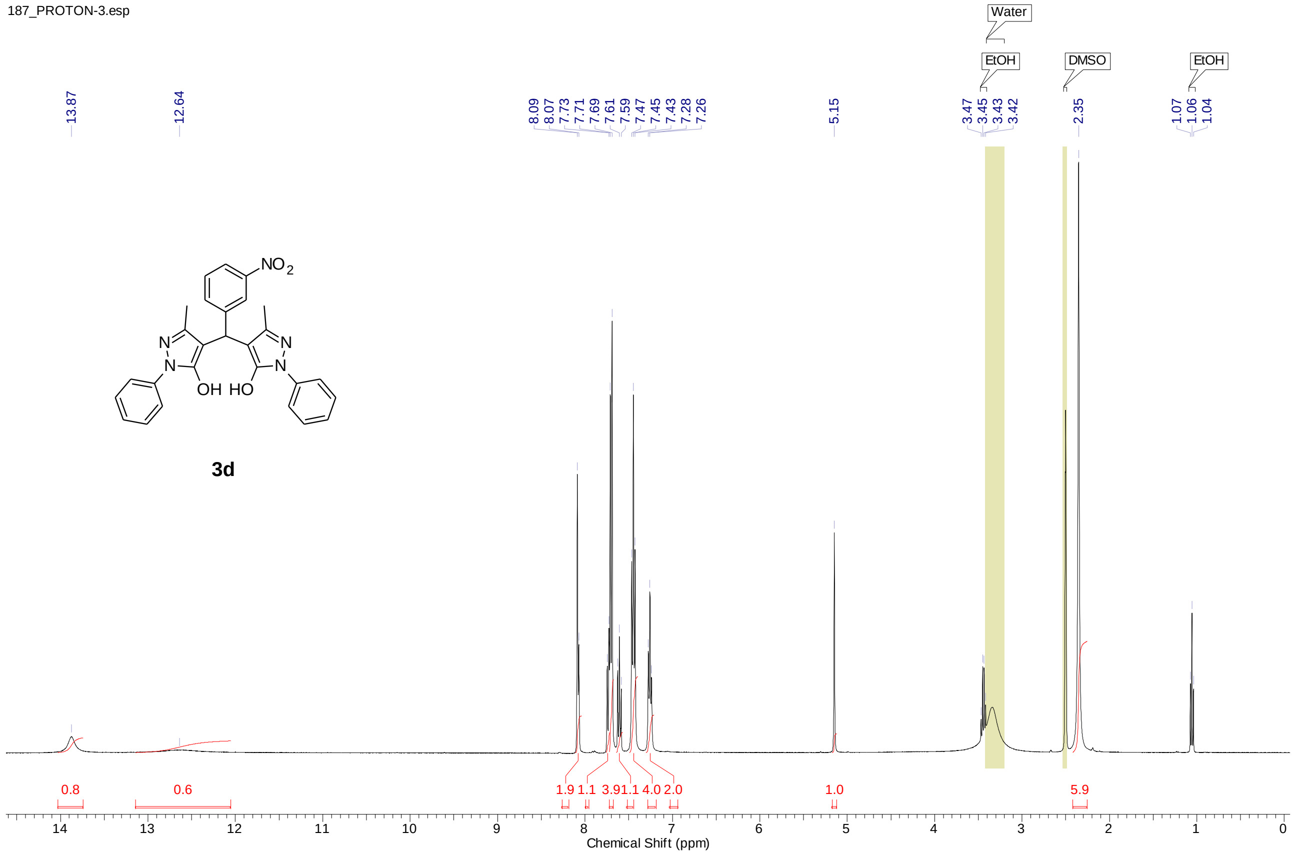

Fig. S5. ¹H NMR spectrum of compound **3d**.

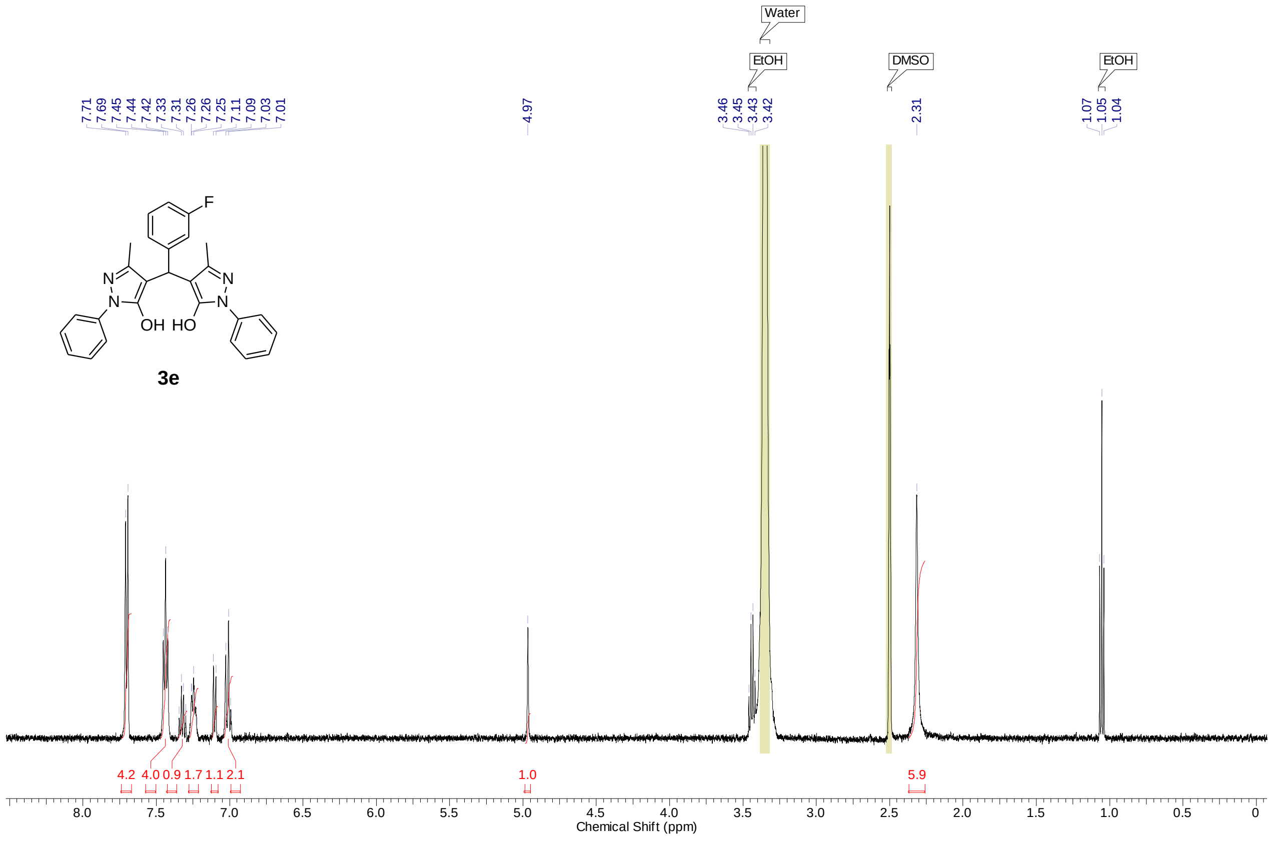

Fig. S6. ^{1}H NMR spectrum of compound **3e**.

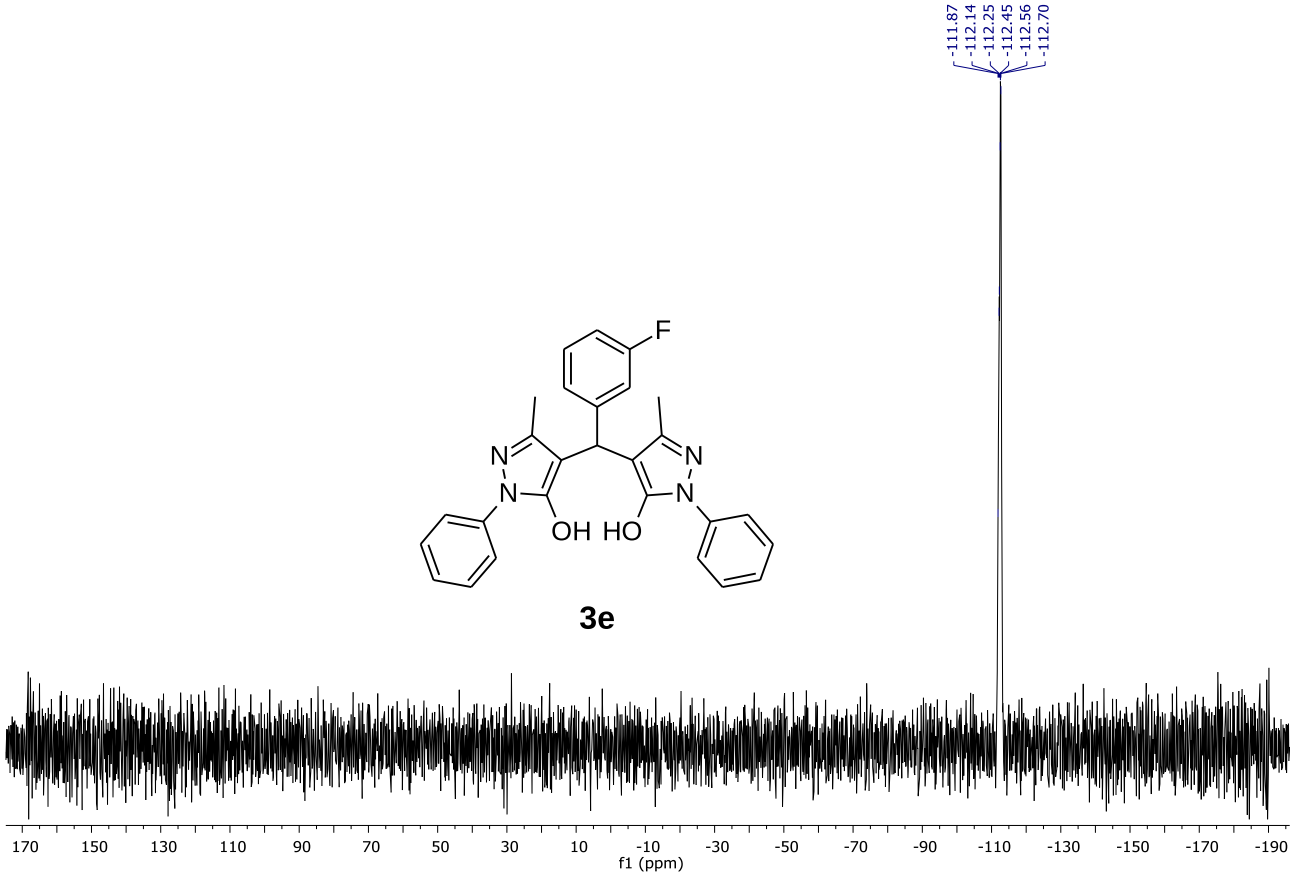

Fig. S7. ^{19}F NMR spectrum of compound **2e**.

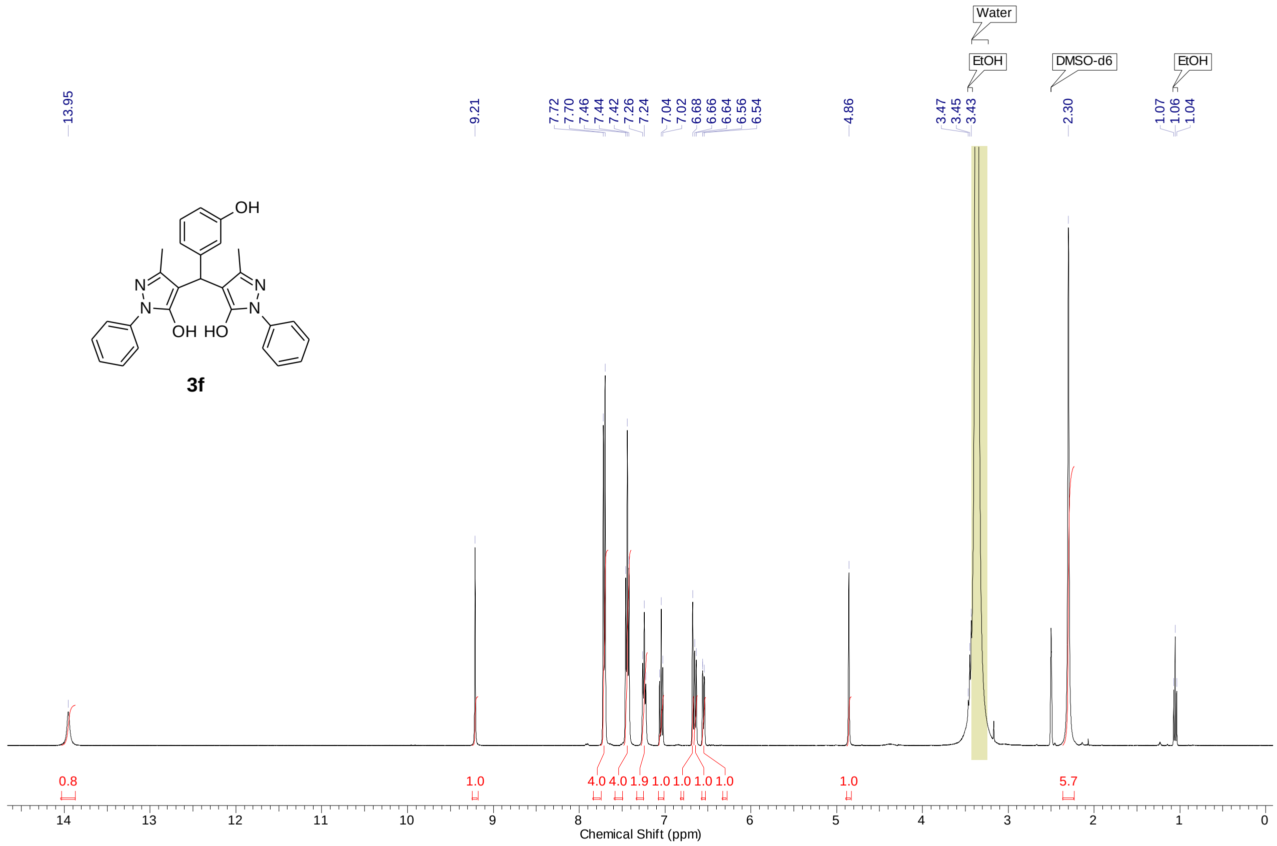

Fig. S8. ¹H NMR spectrum of compound **3f**.

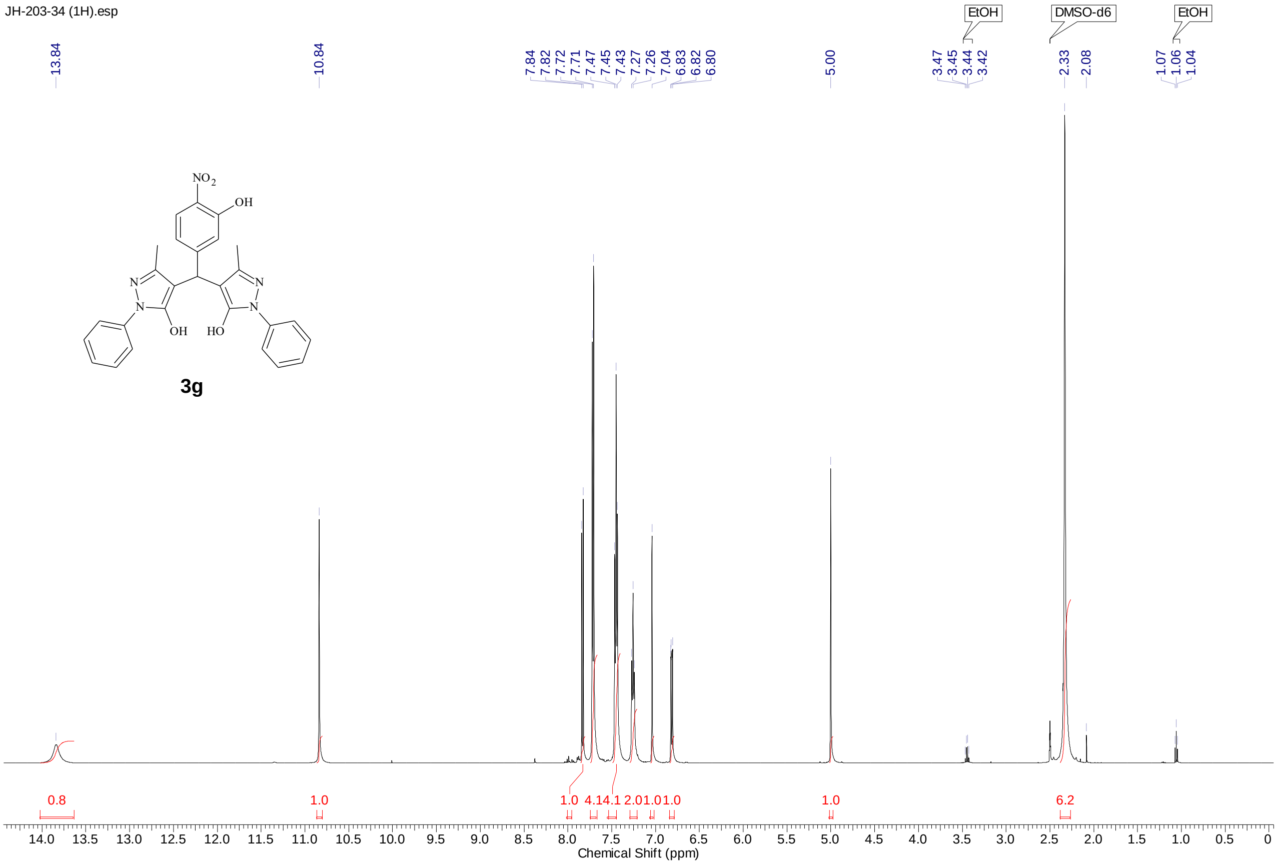

Fig. S9. ¹H NMR spectrum of compound **3g**.

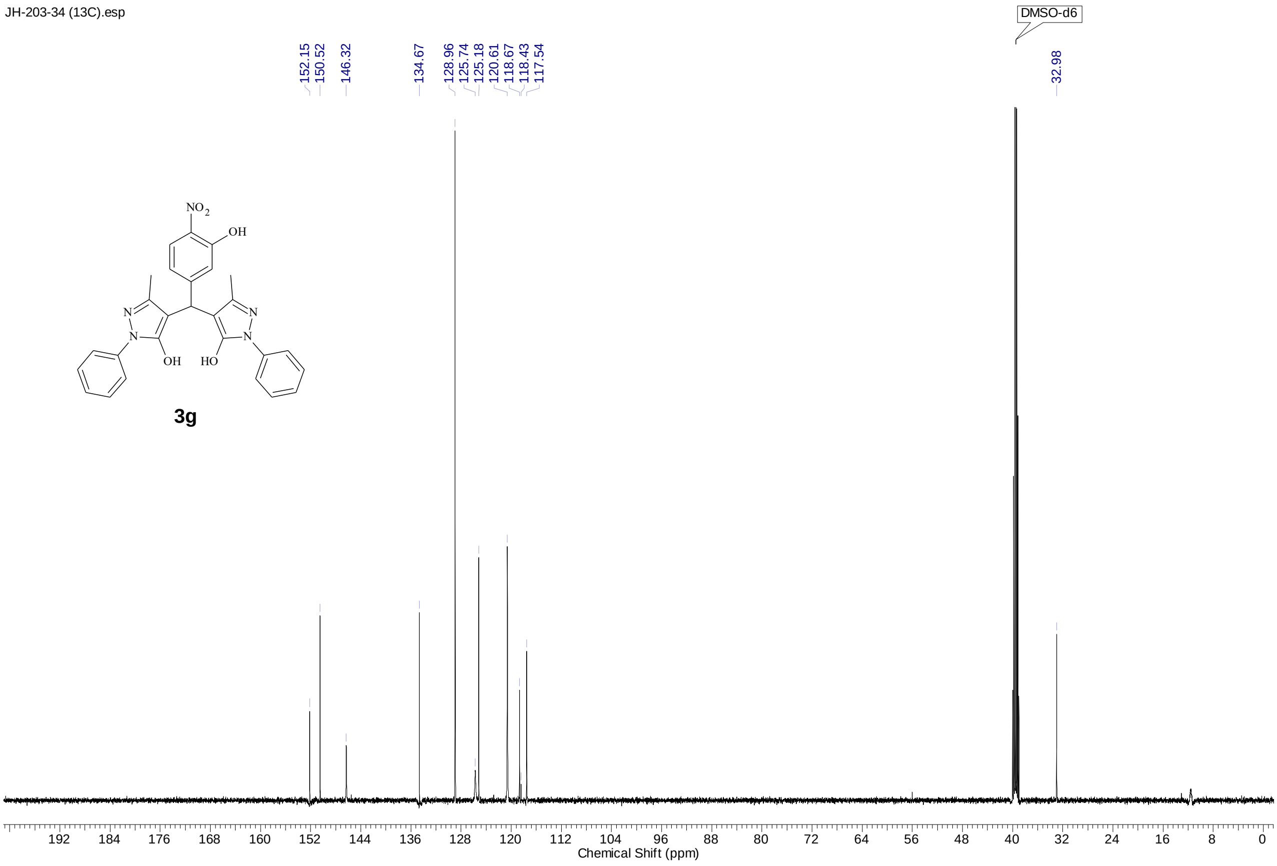

Fig. S10. ^{13}C NMR spectrum of compound **3g**.

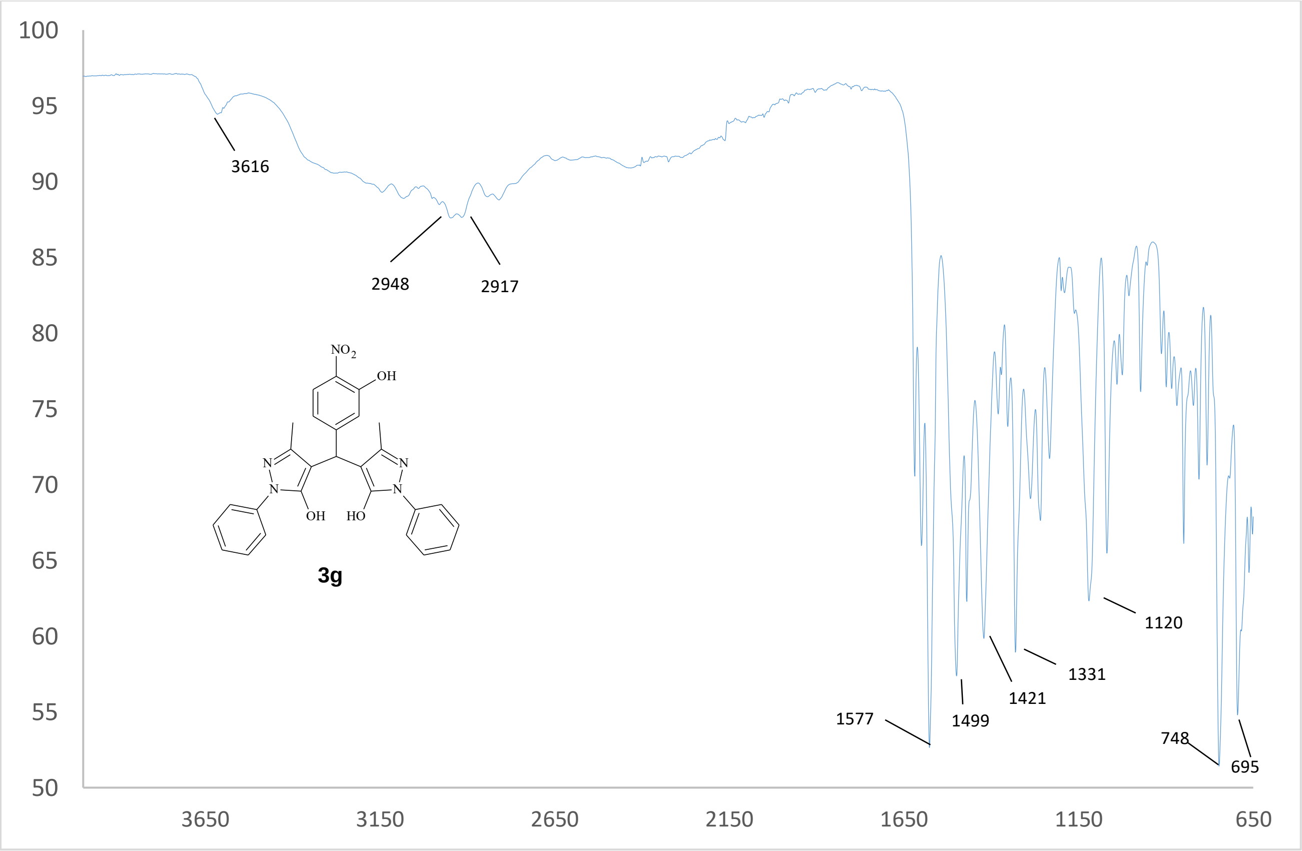

Fig. S11. FTIR spectrum of compound **3g**.

Acquisition Parameter

Ion Source Type	ESI	Ion Polarity	Positive	Alternating Ion Polarity	off
Mass Range Mode	UltraScan	Scan Begin	200 m/z	Scan End	600 m/z
Accumulation Time	2662 µs	RF Level	63 %	Trap Drive	54.1
SPS Target Mass	400 m/z	Averages	5 Spectra	n/a	n/a

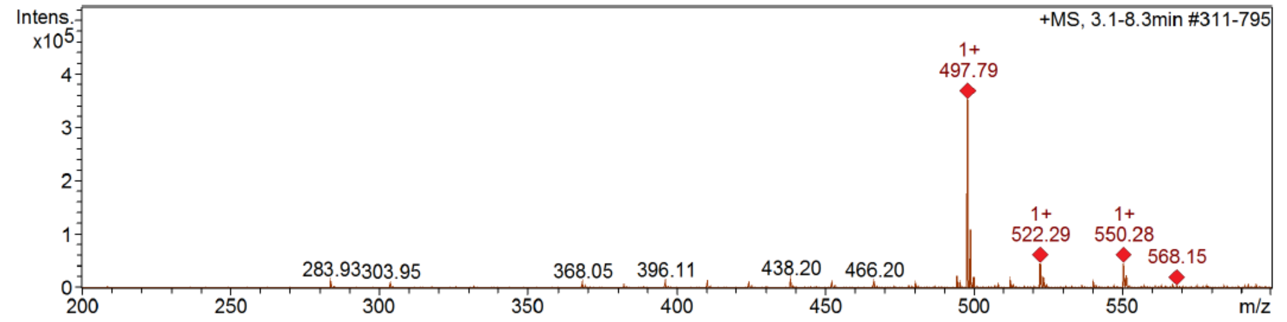

Fig. S12. ESI-MS spectrum of compound **3g**.

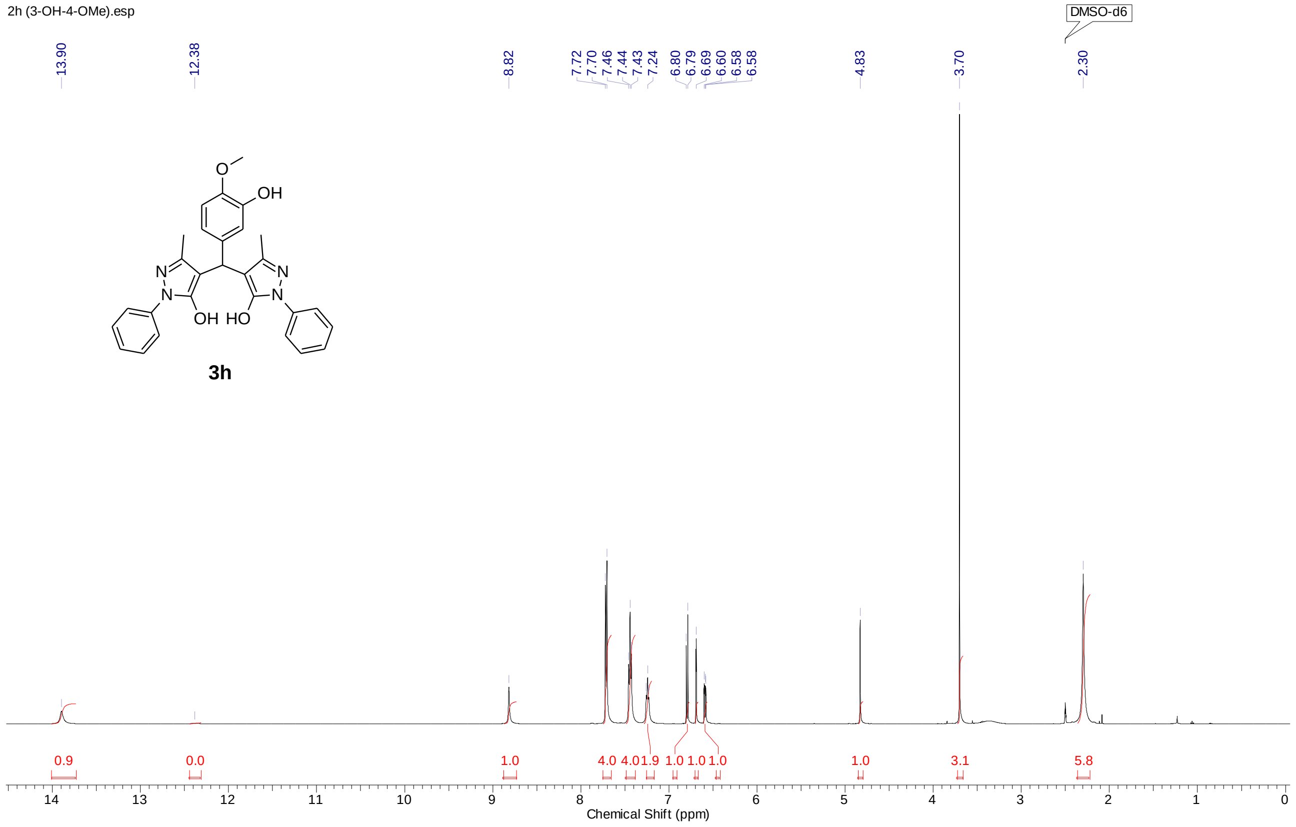

Fig. S13. ^{1}H NMR spectrum of compound **3h**.

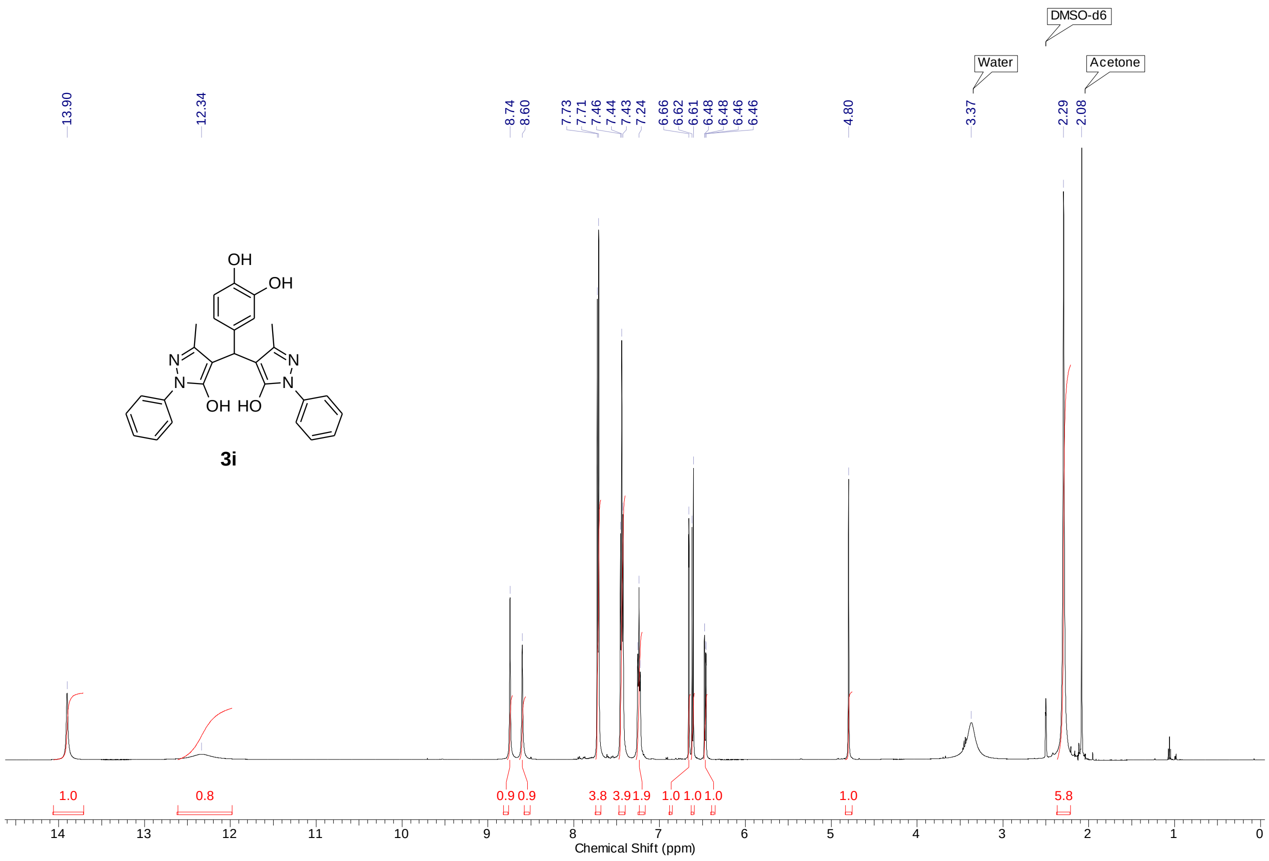

Fig. S14. ^{1}H NMR spectrum of compound **3i**.

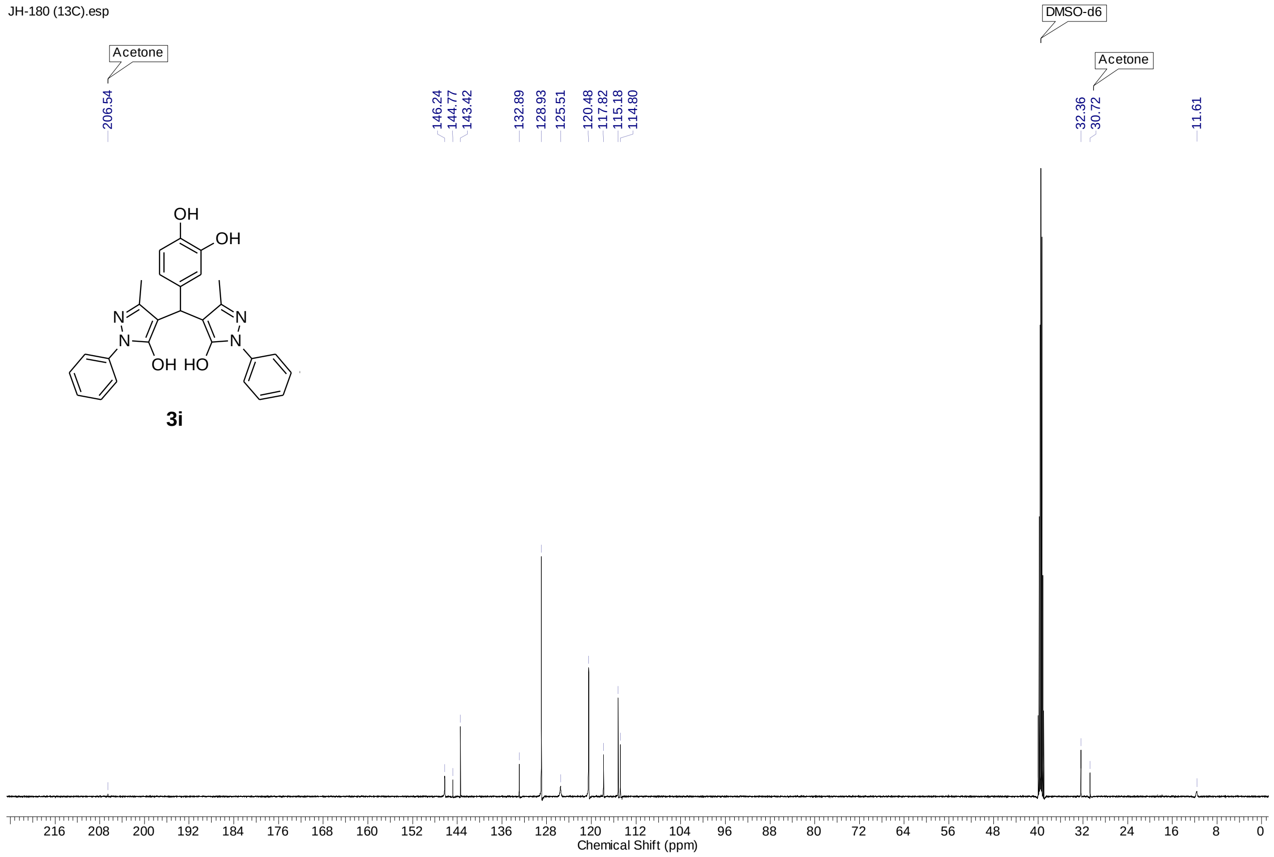

Fig. S15. ^{13}C NMR spectrum of compound **3i**.

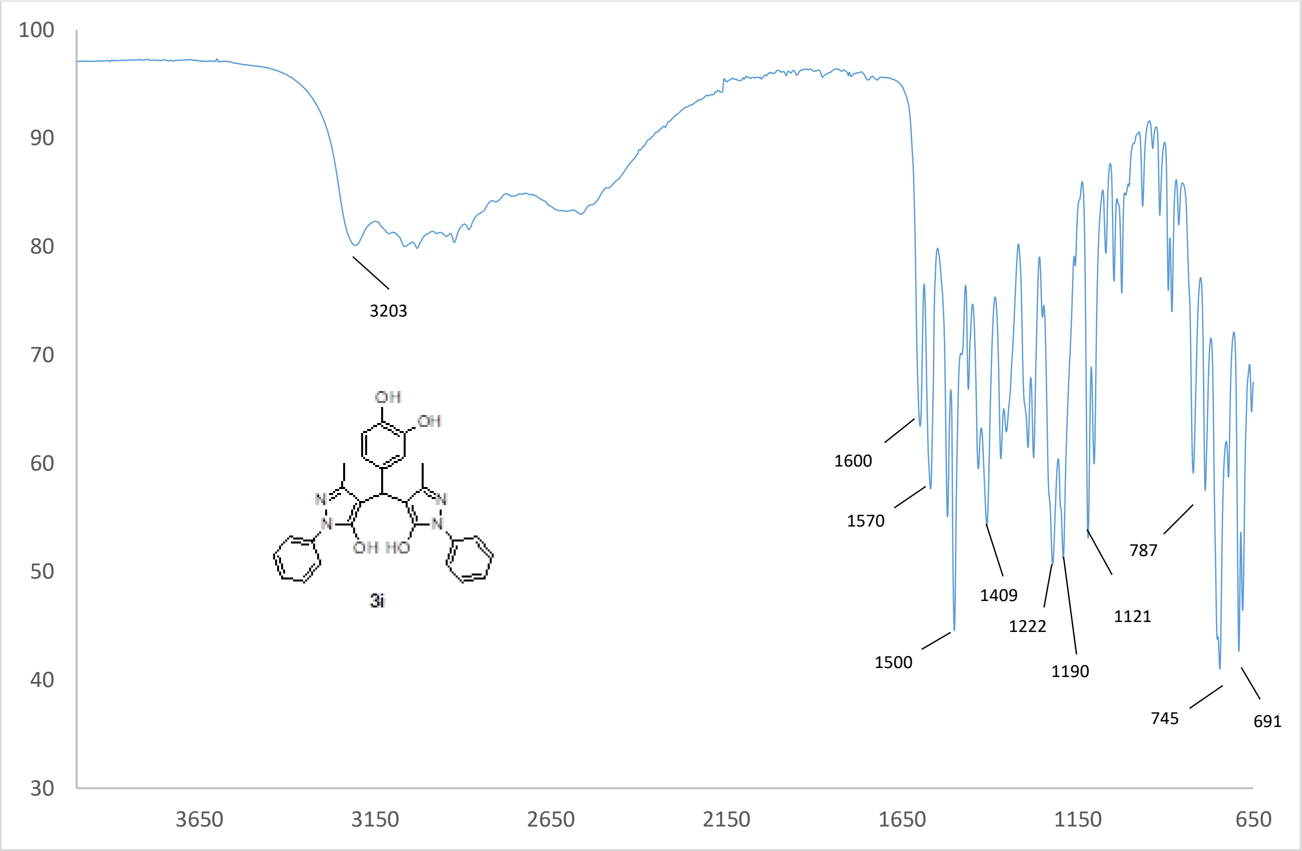

Fig. S16. FTIR spectrum of compound **3i**.

Elemental Composition Report **Page 1**

Single Mass Analysis
Tolerance = 10.0 mDa / DBE: min = -2.0, max = 1000.0
Element prediction: Off
Number of isotope peaks used for i-FIT = 3

Monoisotopic Mass, Even Electron Ions
100 formula(e) evaluated with 3 results within limits (up to 19 closest results for each mass)
Elements Used:
C: 0-100 H: 0-200 N: 4-4 O: 0-30
24-Jun-2016
jhm-24jun16-180 212 (3.921) Cn (Cen,7, 50.00, Ar); Sm (SG, 3x5.00); Sb (12,5.00) TOF MS ES+ 6.58e+003

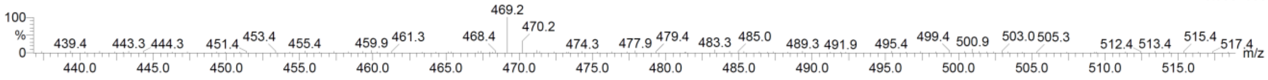

Mass	Calc. Mass	mDa	PPM	DBE	i-FIT	Formula
Minimum:				-2.0		
Maximum:		10.0	10.0	1000.0		
469.1877	469.1876	0.1	0.2	17.5	8.3	C27 H25 N4 O4
	469.1935	-5.8	-12.4	8.5	70.8	C20 H29 N4 O9
	469.1782	9.5	20.2	4.5	172.4	C16 H29 N4 O12

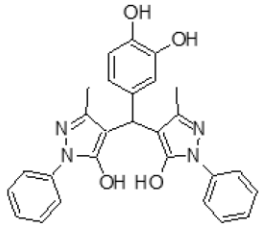

Molecular Formula = $C_{27}H_{24}N_4O_4$
[M+H]+ = 469.187032 Da

Fig. S17. HRMS spectrum of compound **3i**.

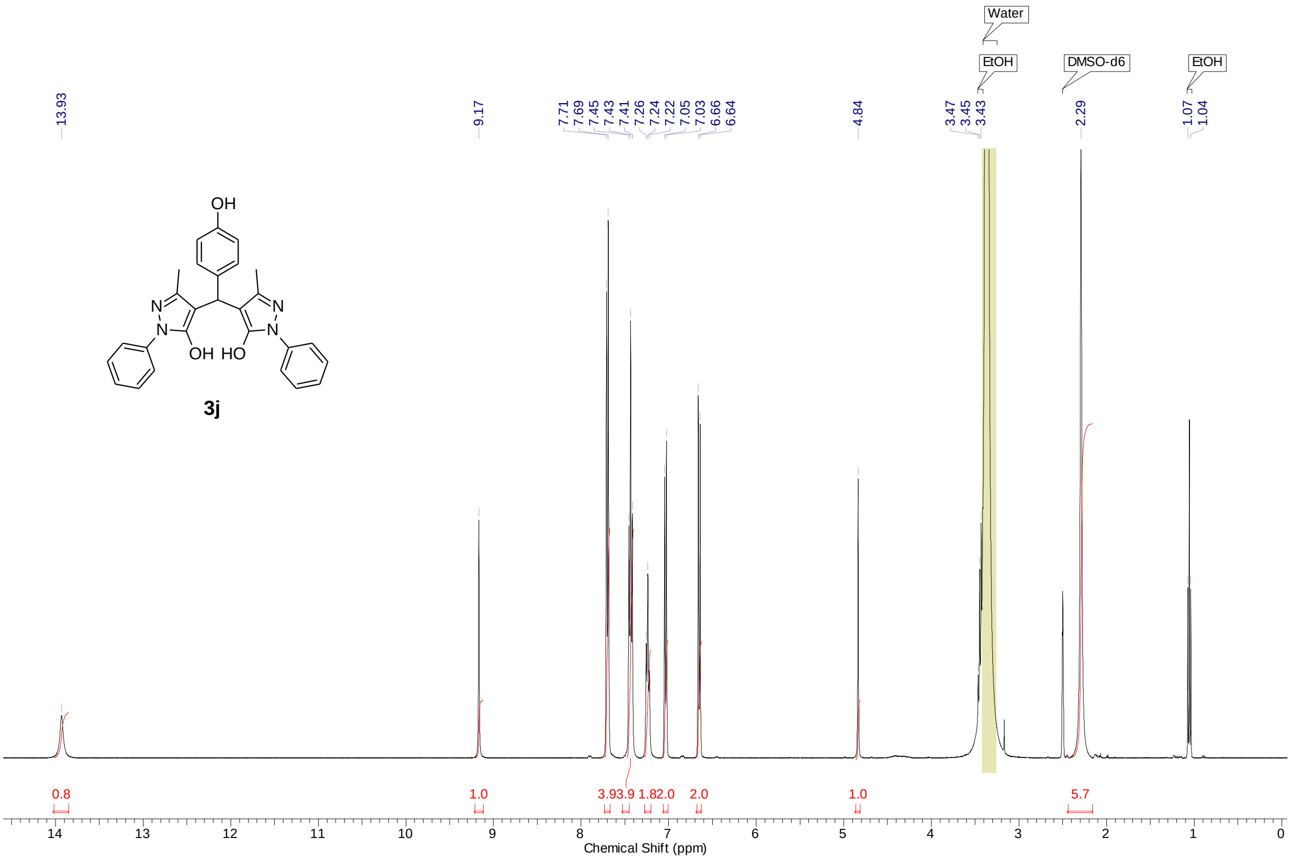

Fig. S18. ¹H NMR spectrum of compound **3j**.

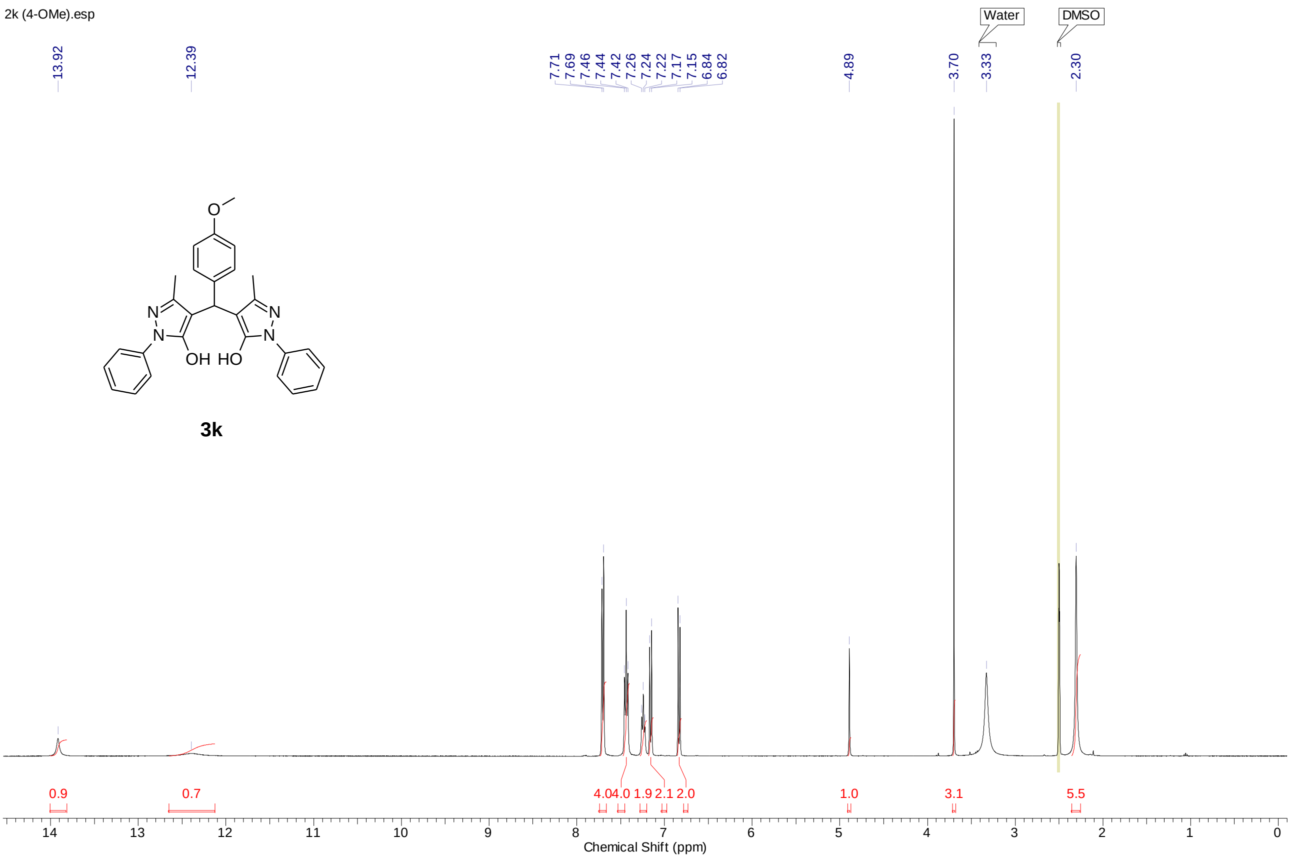

Fig. S19. ¹H NMR spectrum of compound **3k**.

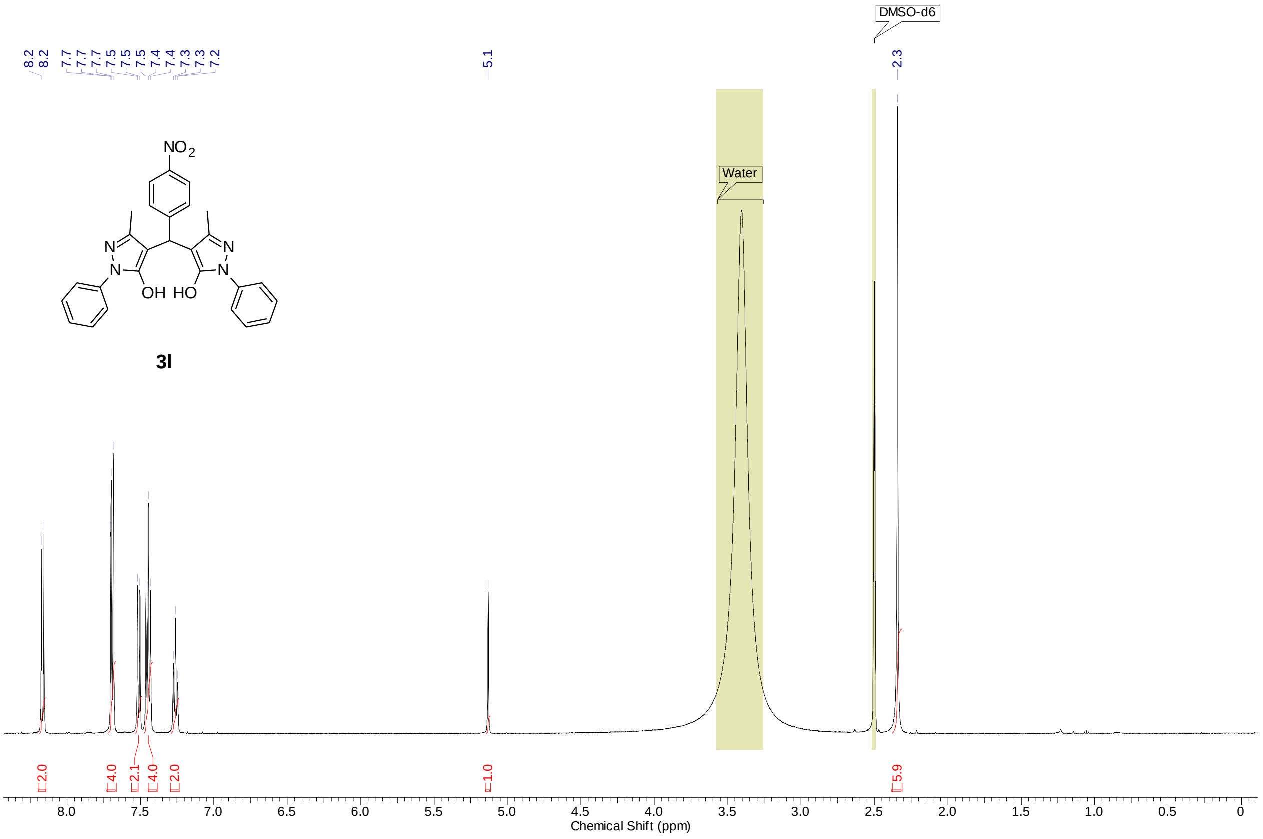

Fig. S20. ^{1}H NMR spectrum of compound **3l**.

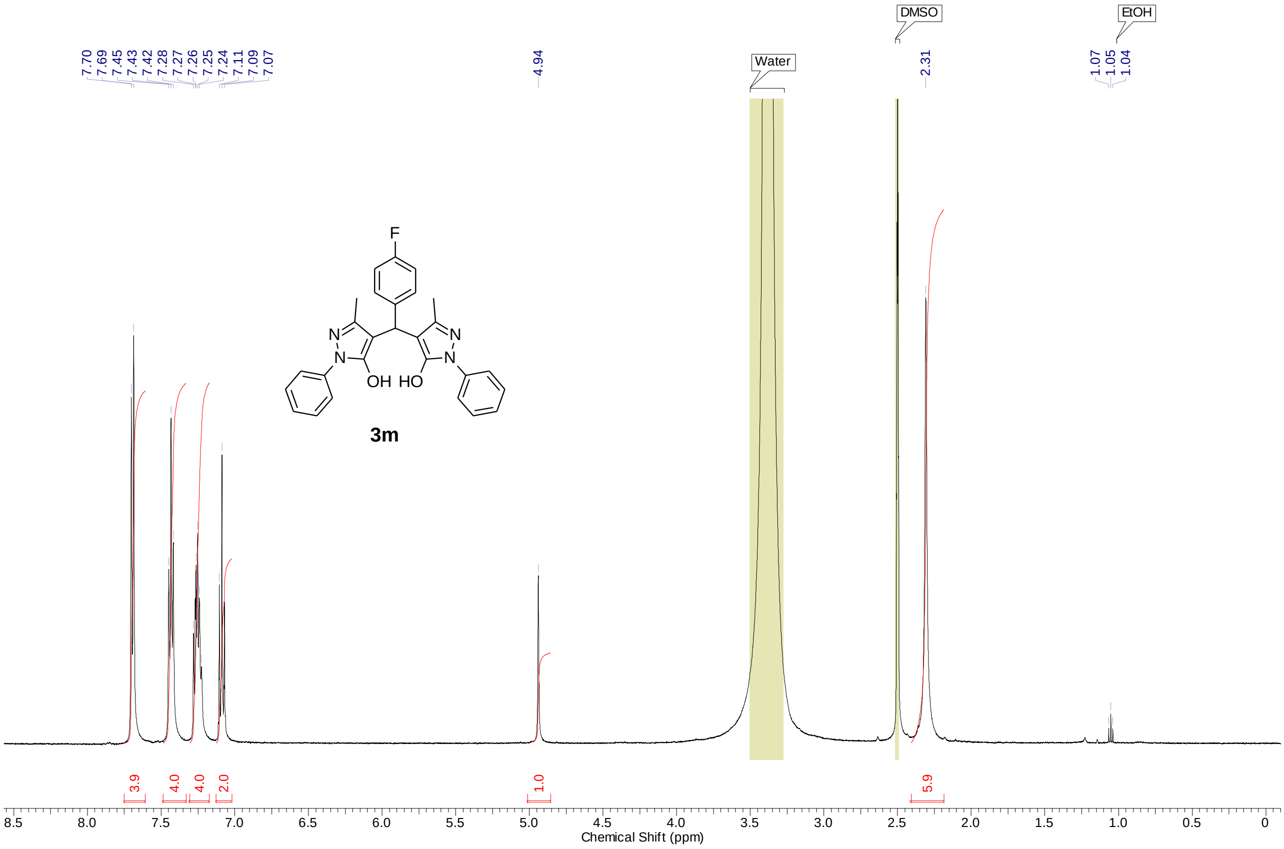

Fig. S21. ^{1}H NMR spectrum of compound **3m**.

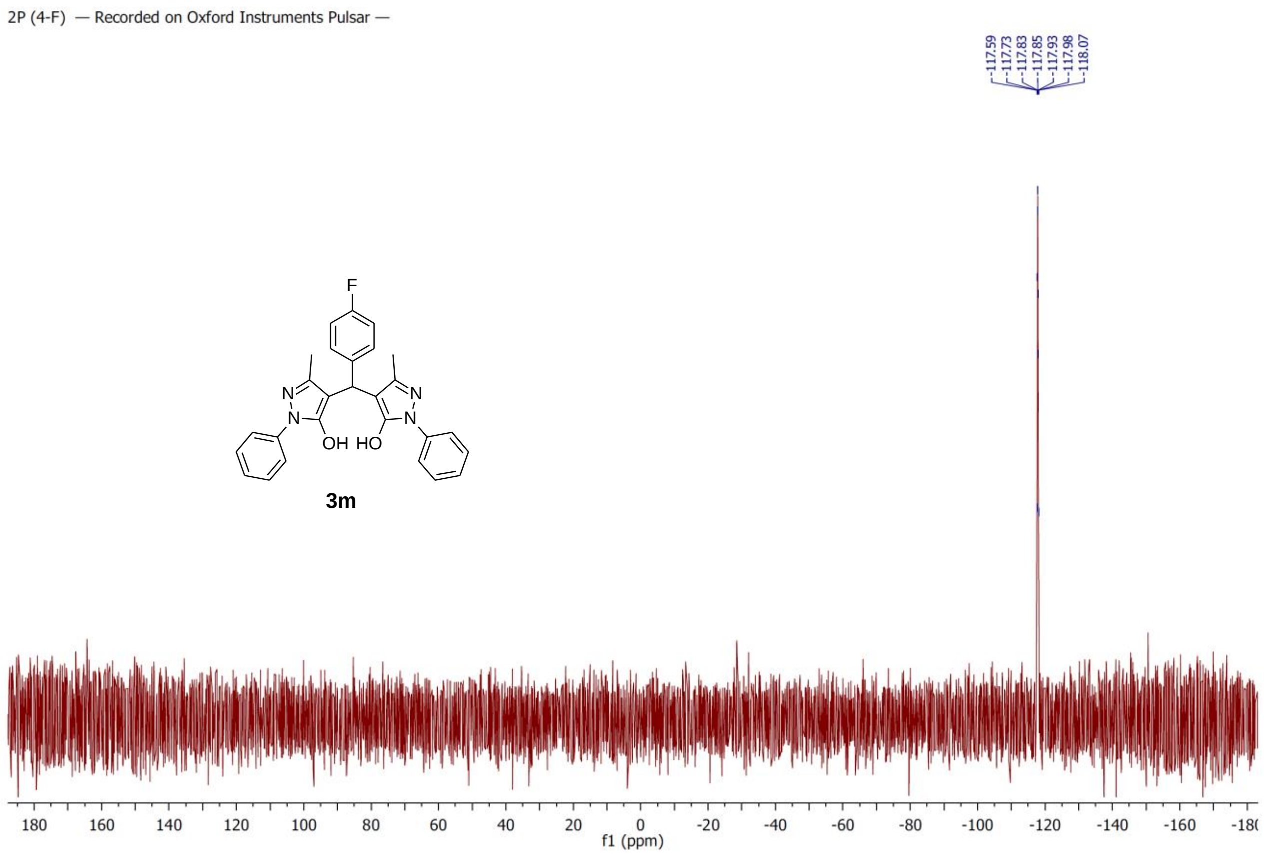

Fig. S22. ^{19}F NMR spectrum of compound **3m**.

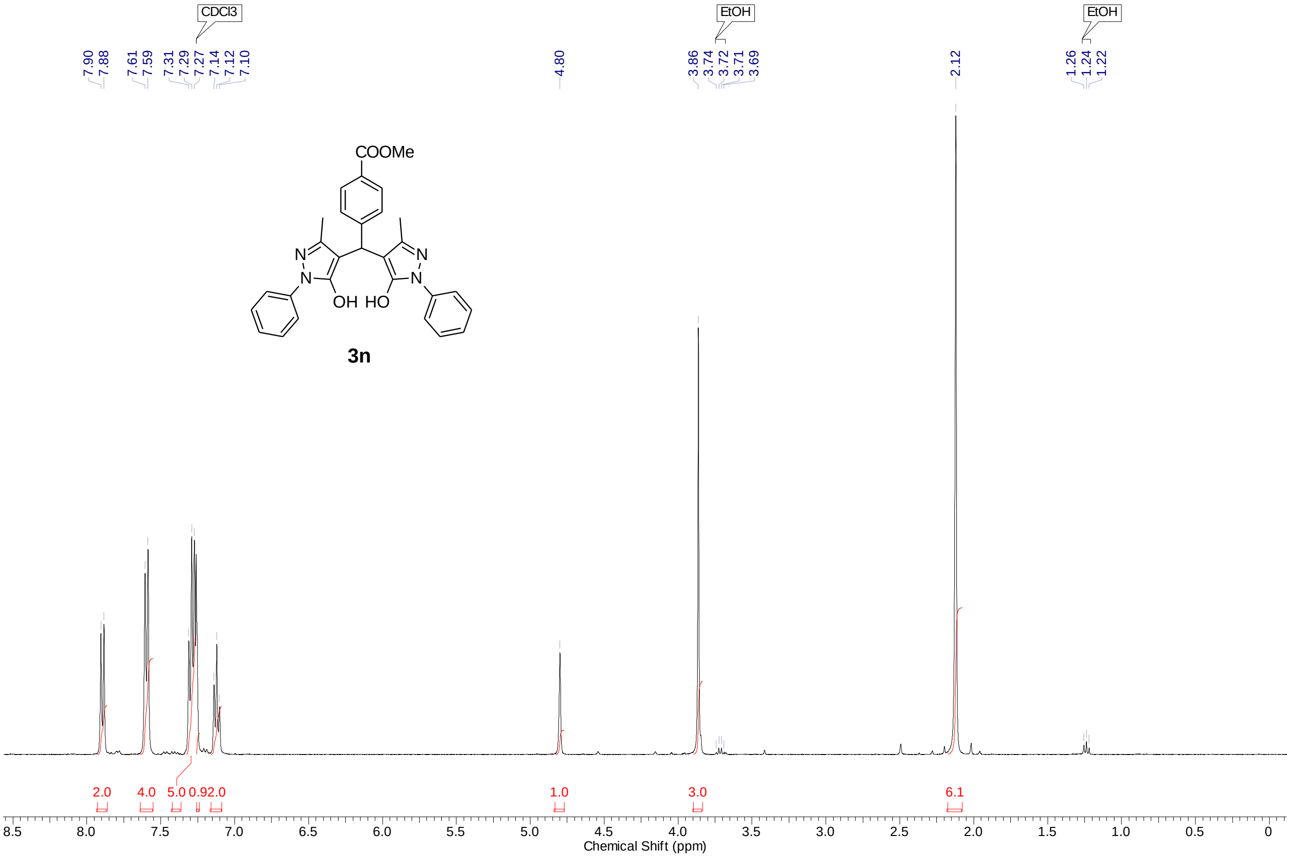

Fig. S23. ^{1}H NMR spectrum of compound **3n**.

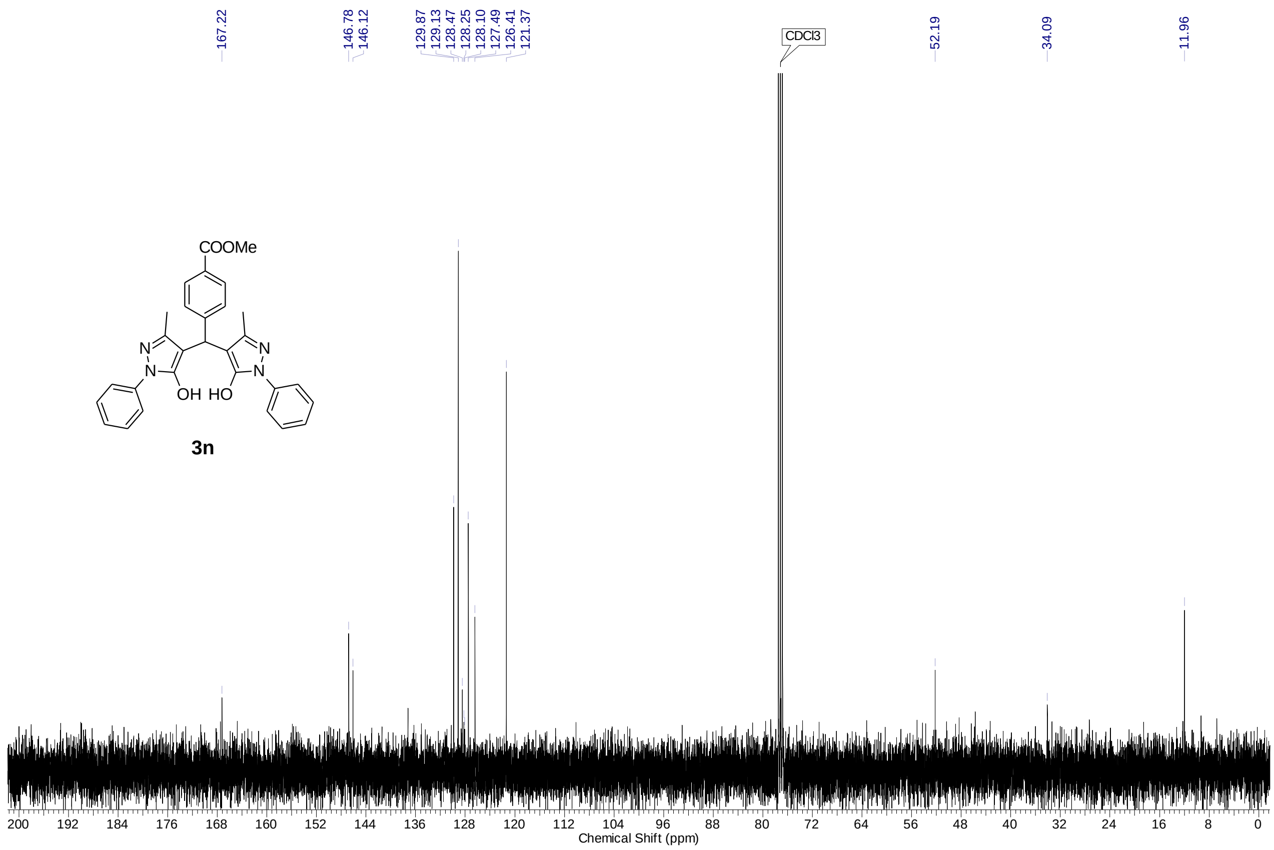

Fig. S24. ^{13}C NMR spectrum of compound **3n**.

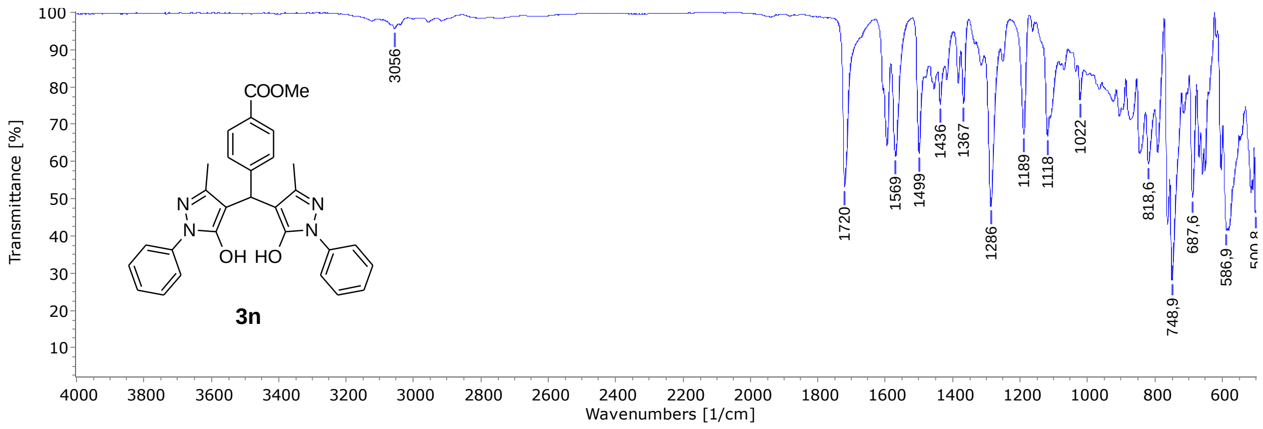

Fig. S25. FTIR spectrum of compound **3n**.

Acquisition Parameter

Ion Source Type	ESI	Ion Polarity	Positive	Alternating Ion Polarity	off
Mass Range Mode	UltraScan	Scan Begin	200 m/z	Scan End	600 m/z
Accumulation Time	100000 µs	RF Level	63 %	Trap Drive	54.1
SPS Target Mass	400 m/z	Averages	5 Spectra	n/a	n/a

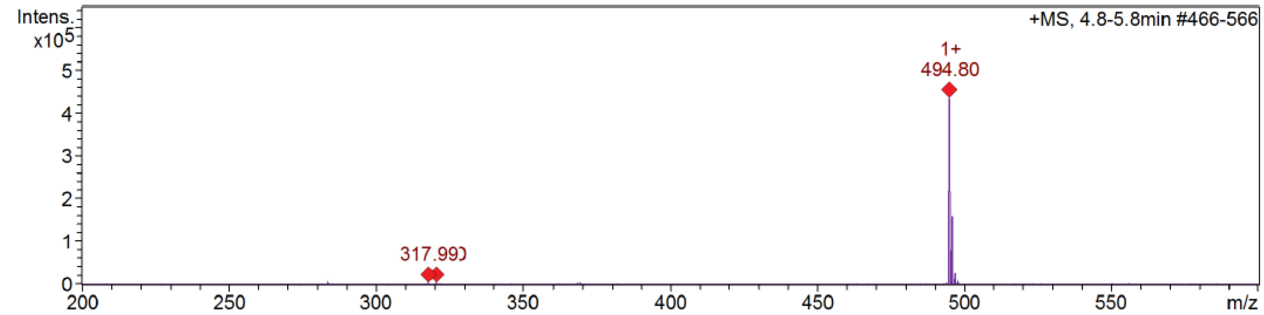

Fig. S26. ESI-MS spectrum of compound **3n**.

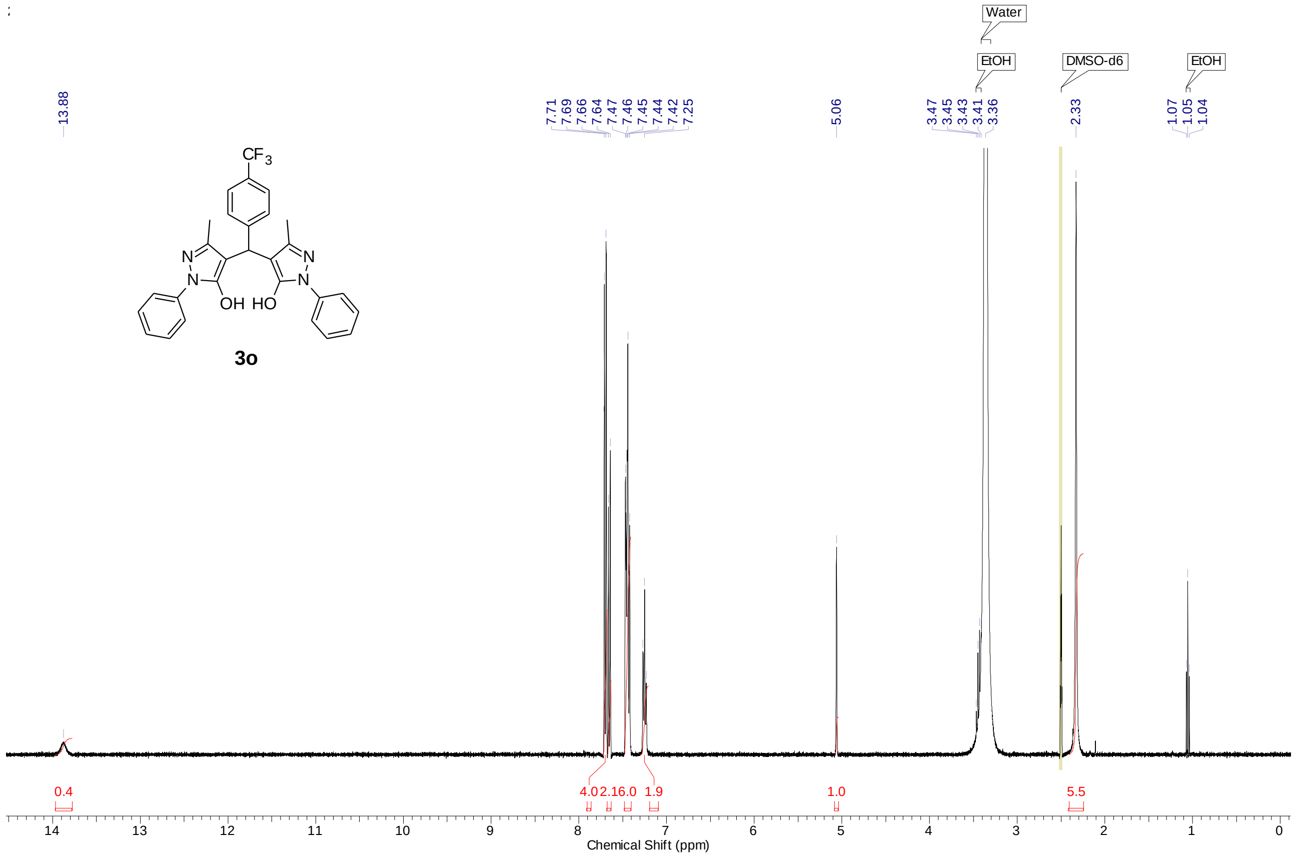

Fig. S27. ^{1}H NMR spectrum of compound **3o**.

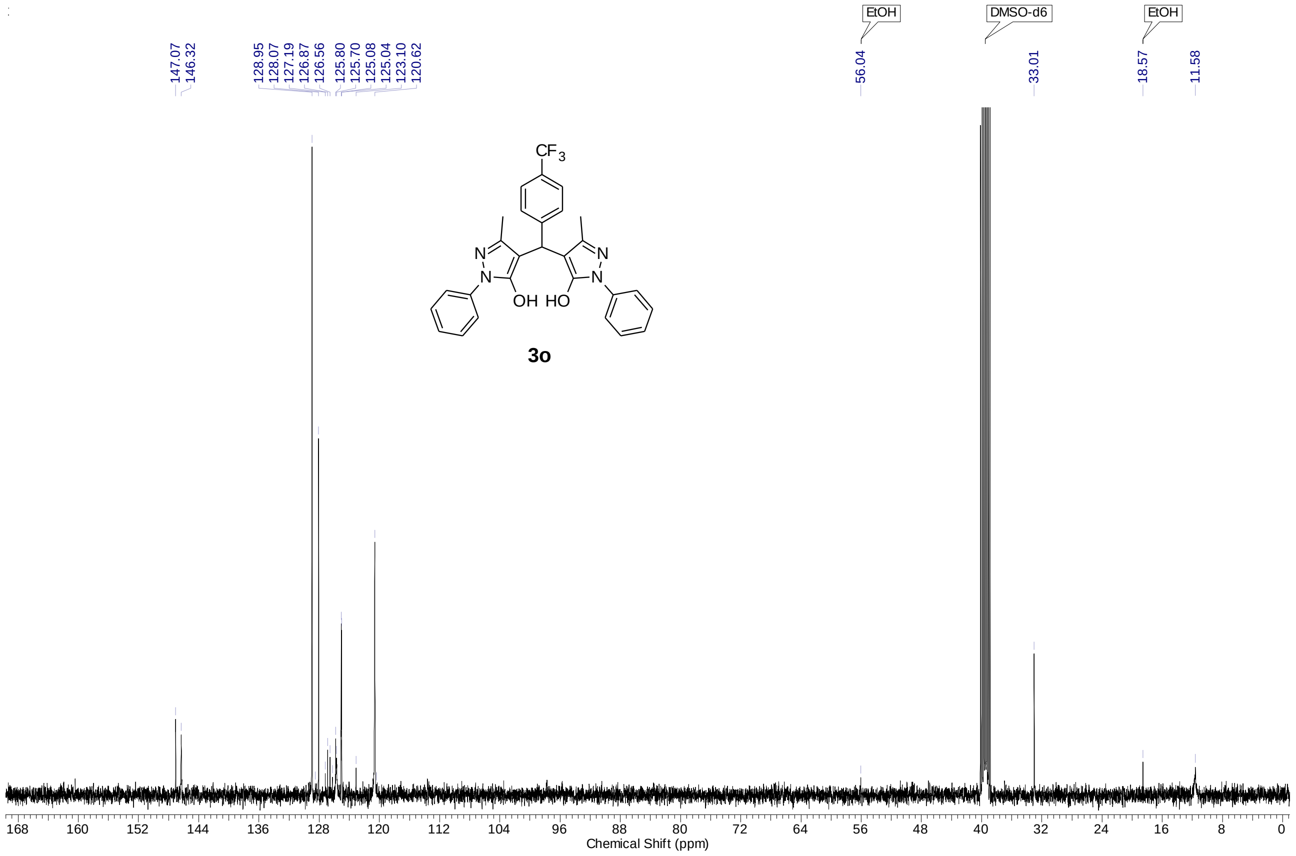

Fig. S28. ^{13}C NMR spectrum of compound **3o**.

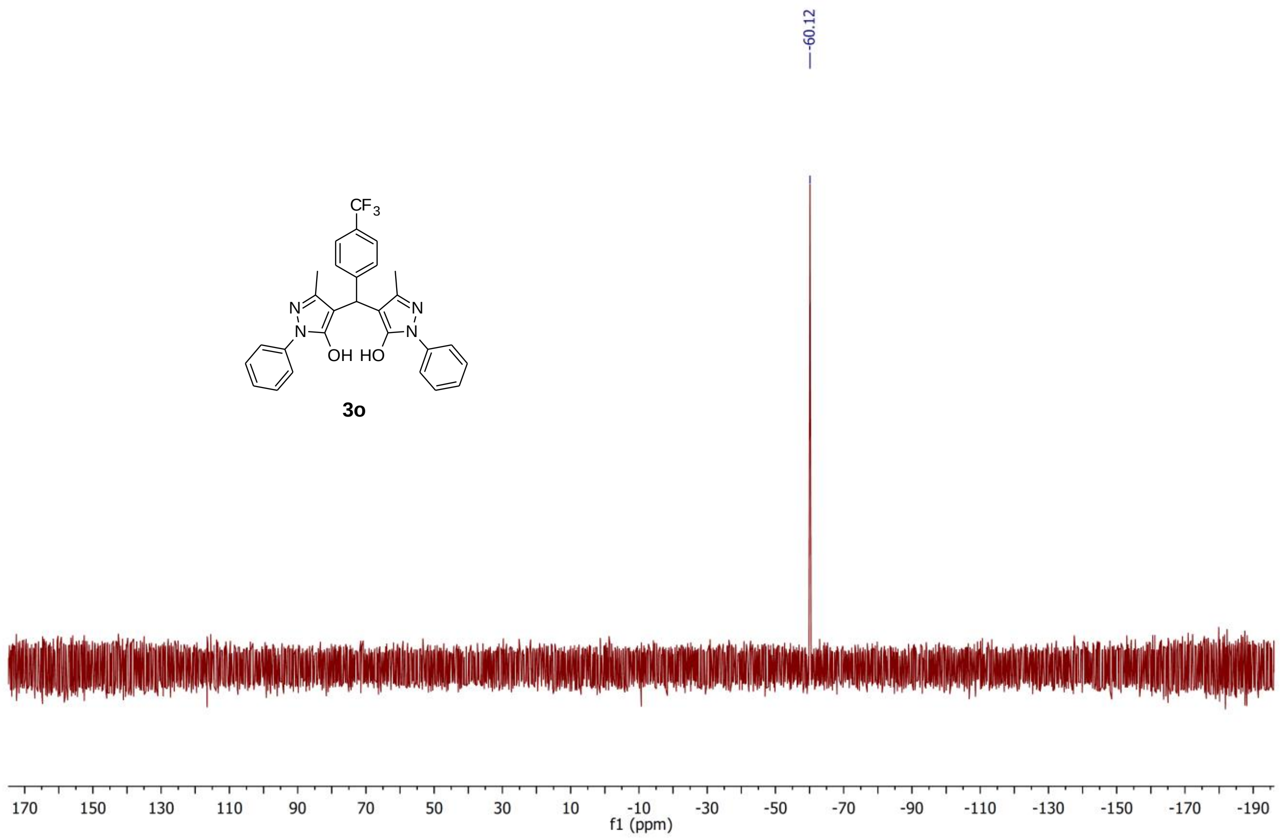

Fig. S29. ^{19}F NMR spectrum of compound **3o**.

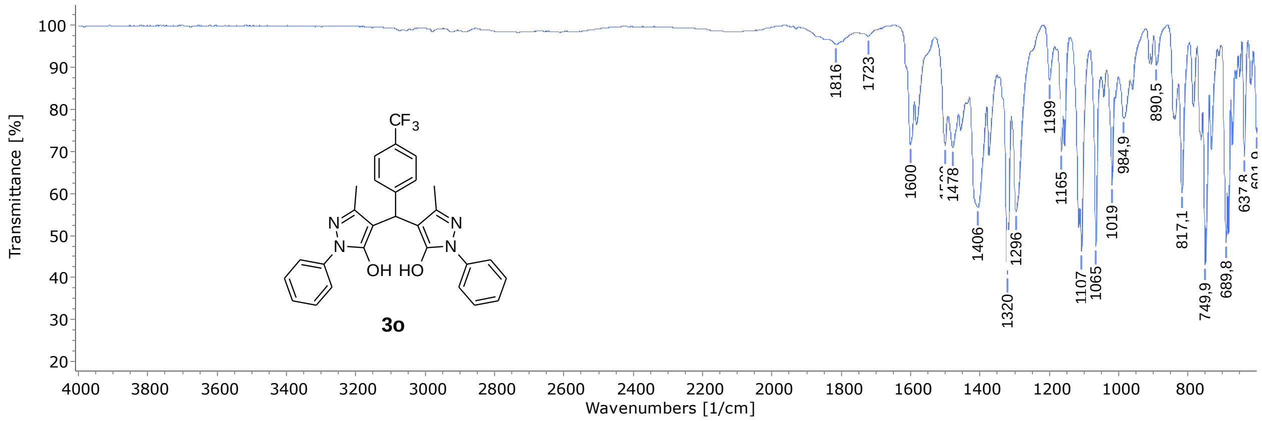

Fig. S30. FTIR spectrum of compound **3o**.

Acquisition Parameter

Ion Source Type	ESI	Ion Polarity	Positive	Alternating Ion Polarity	off
Mass Range Mode	UltraScan	Scan Begin	200 m/z	Scan End	600 m/z
Accumulation Time	517 µs	RF Level	63 %	Trap Drive	54.1
SPS Target Mass	400 m/z	Averages	5 Spectra	n/a	n/a

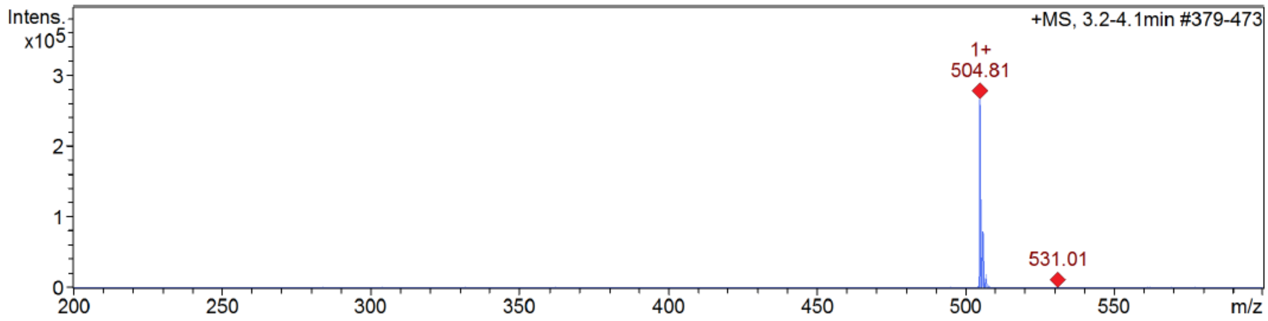

Fig. S31. ESI-MS spectrum of compound **3o**.

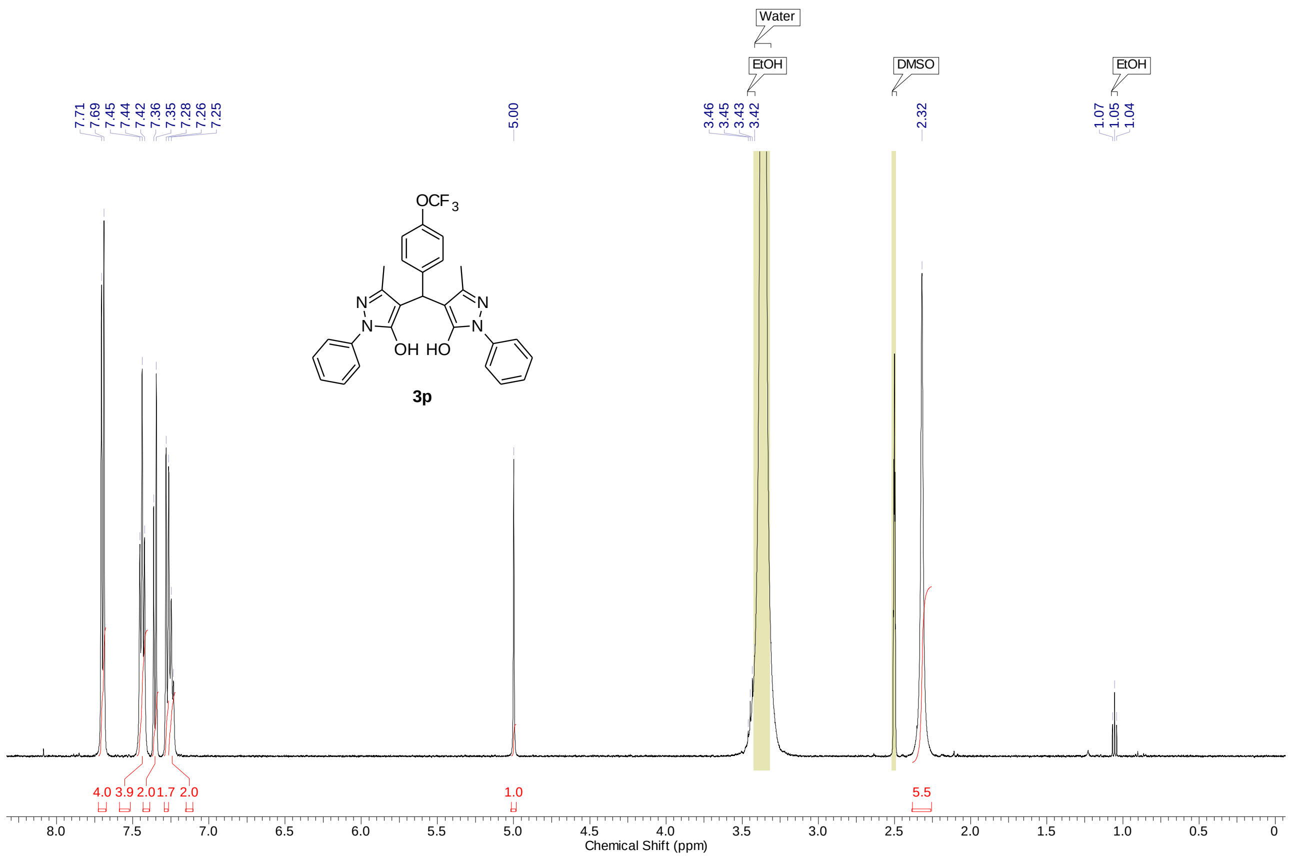

Fig. S32. ^{1}H NMR spectrum of compound **3p**.

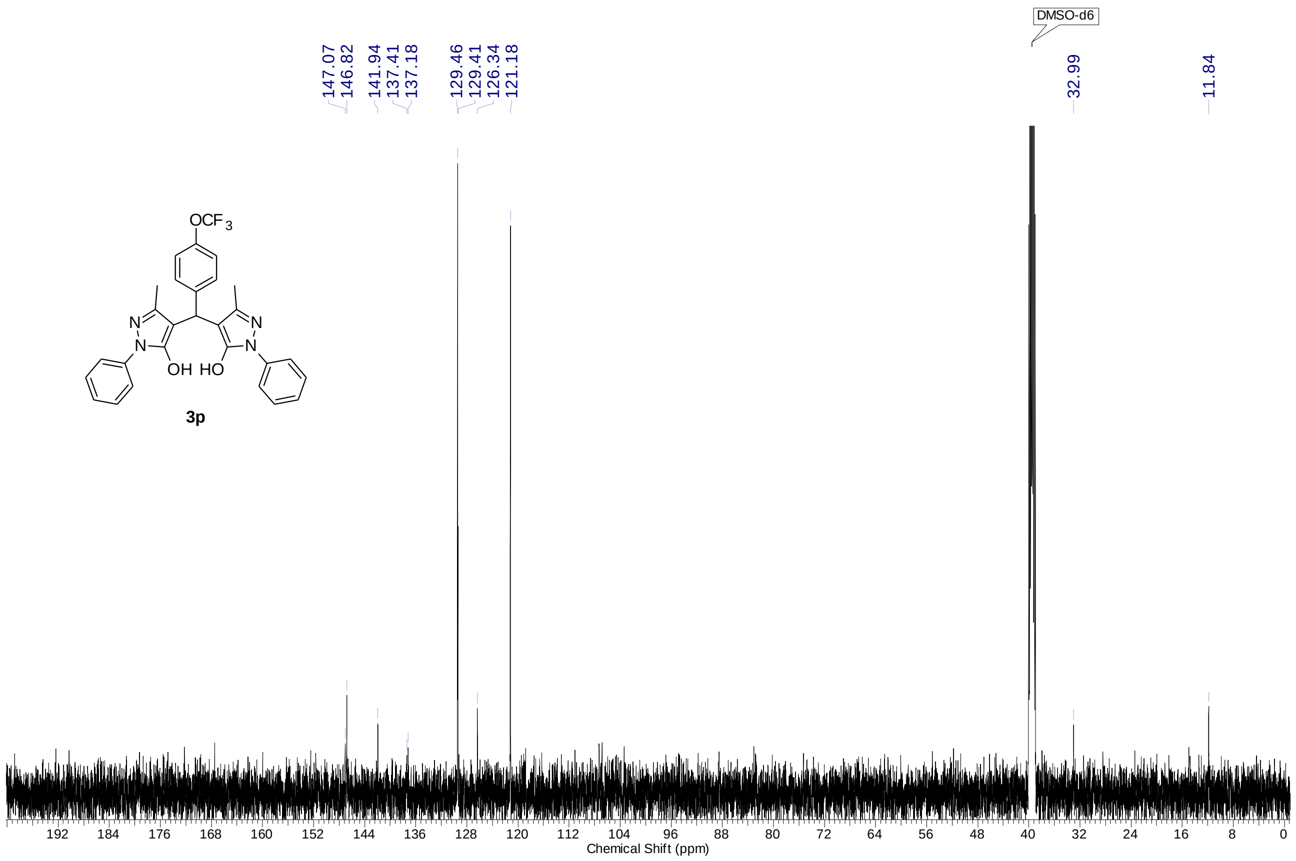

Fig. S33. ^{13}C NMR spectrum of compound **3p**.

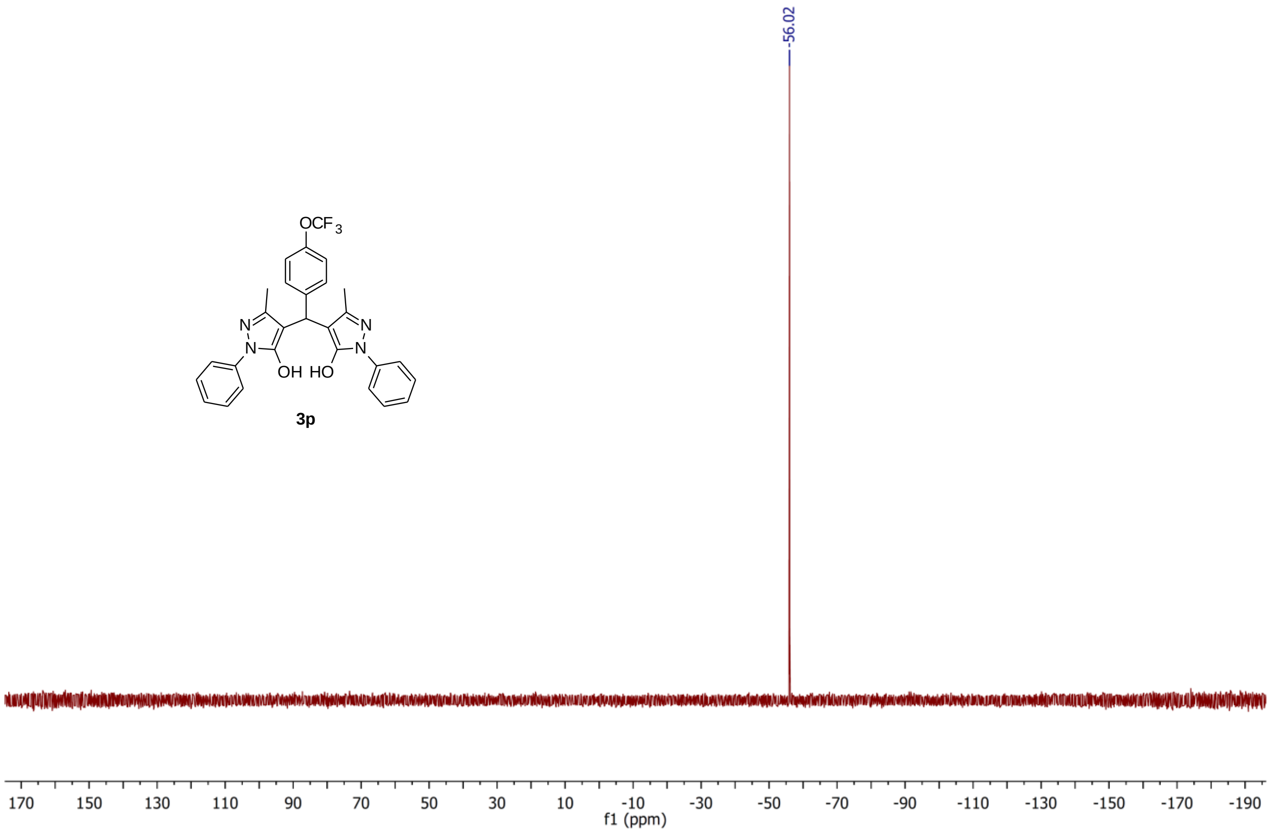

Fig. S34. ^{19}F NMR spectrum of compound **3p**.

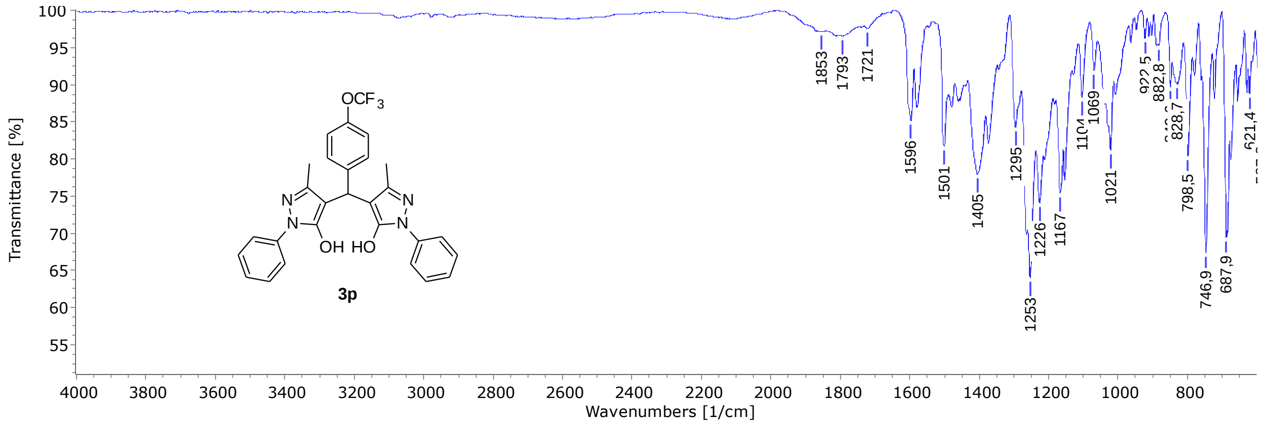

Fig. S35. FTIR spectrum of compound **3p**.

Acquisition Parameter

Ion Source Type	ESI	Ion Polarity	Positive	Alternating Ion Polarity	off
Mass Range Mode	UltraScan	Scan Begin	200 m/z	Scan End	600 m/z
Accumulation Time	1209 µs	RF Level	63 %	Trap Drive	54.1
SPS Target Mass	400 m/z	Averages	5 Spectra	n/a	n/a

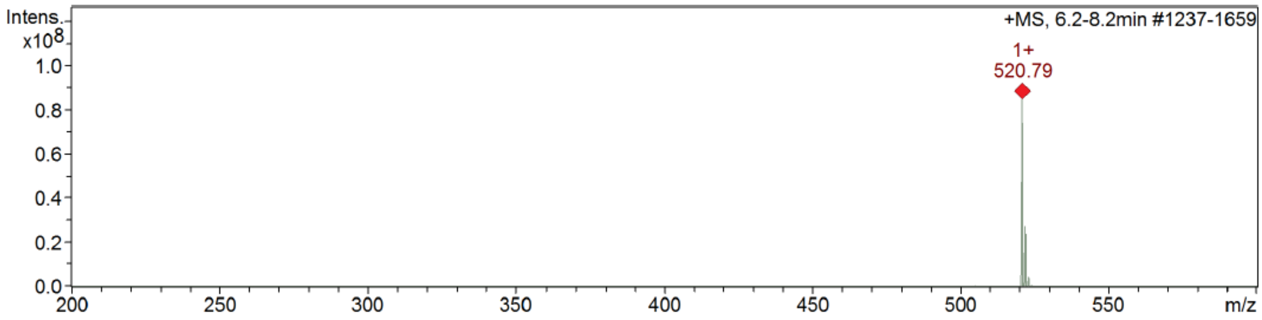

Fig. S36. ESI-MS spectrum of compound **3p**.

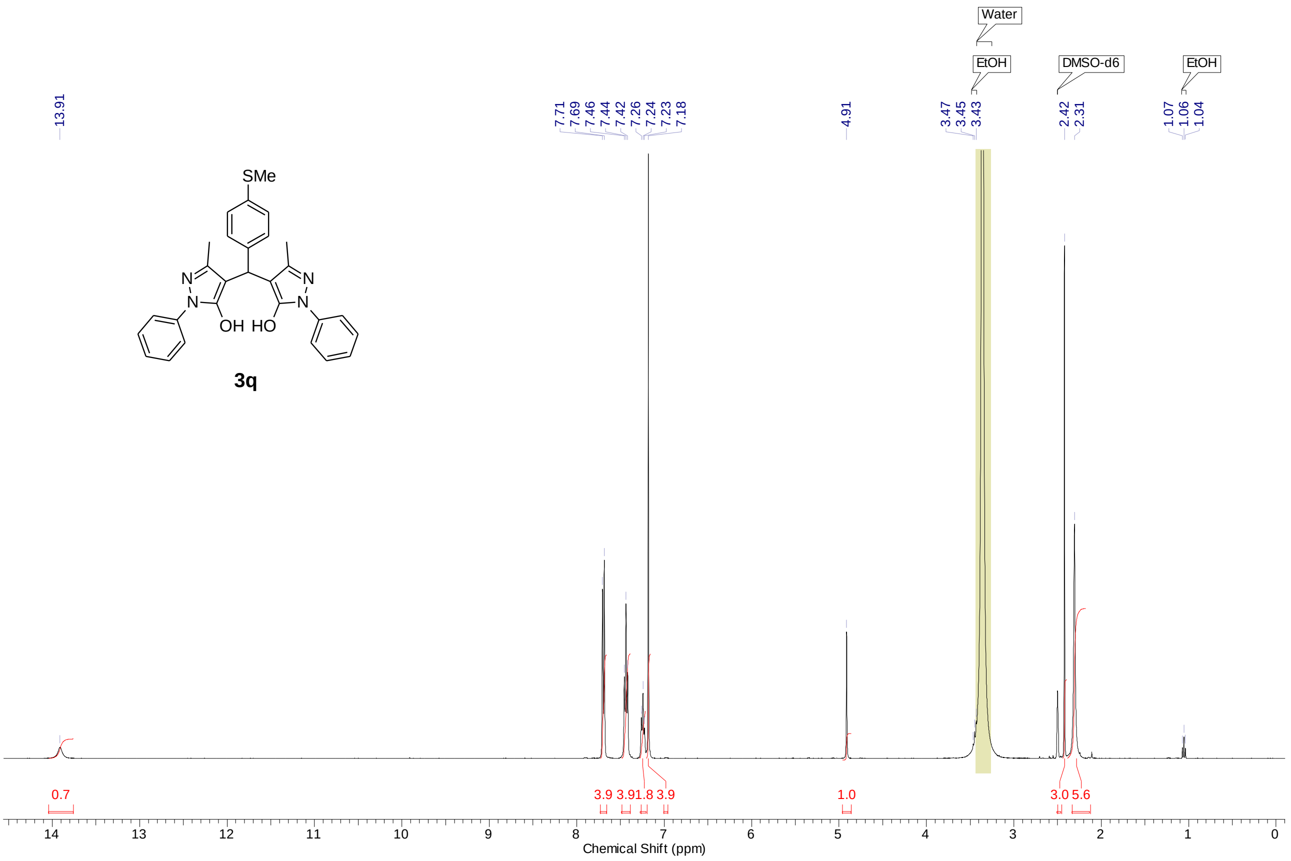

Fig. S37. ^{1}H NMR spectrum of compound **3q**.
